# Electro-oxidation sensing of sumatriptan in aqueous solutions and human blood serum by Zn(II)-MOF modified electrochemical delaminated pencil graphite electrode

**DOI:** 10.1038/s41598-023-44034-5

**Published:** 2023-10-05

**Authors:** Lotfali Saghatforoush, Tohid Mahmoudi, Zeynab Khorablou, Hassan Nasiri, Akbar Bakhtiari, Seyed Ali Akbar Sajadi

**Affiliations:** 1 Department of Chemistry, Payame Noor University, P.O. Box 19395-4697, Tehran, Iran; 2 Sharif Energy, Water and Environment Institute (SEWEI), Sharif University of Technology, P.O. Box 11155-8639, Tehran, Iran; 3 Department of Electrical and Computer Engineering, University of Tabriz, Tabriz, Iran

**Keywords:** Electrochemistry, Inorganic chemistry, Materials chemistry, Chemistry, Analytical chemistry, Sensors, Materials for devices, Sensors and biosensors

## Abstract

An electrochemical sensory platform is presented for determination of sumatriptan (SUM) in aqueous solutions and human blood serum. A pencil graphite electrode (PGE) was electrochemically delaminated by cyclic voltammetry technique, and then further modified using nanoparticles of a zinc-based metal–organic framework (Zn(II)-MOF). The fabricated Zn(II)-MOF/EDPGE electrode was utilized for sensitive electrochemical detection of SUM via an electro-oxidation reaction. The Zn(II)-MOF was hydrothermally synthesized and characterized by various techniques. The electrochemical delamination of PGE results in a porous substrate, facilitating the effective immobilization of the modifier. The designed sensor benefits from both enhanced surface area and an accelerated electron transfer rate, as evidenced by the chronocoulogram and Nyquist plots. Under optimized conditions, the developed sensor exhibited a linear response for 0.99–9.52 µM SUM solutions. A short response time of 5 s was observed for the fabricated sensor and the detection limit was found to be 0.29 μM. Selectivity of Zn(II)-MOF/EDPGE towards SUM was evaluated by examining the interference effect of codeine, epinephrine, acetaminophen, ascorbic acid, and uric acid, which are commonly found in biological samples. The developed sensor shows excellent performance with recovery values falling within the range of 96.6 to 111% for the analysis of SUM in human blood serum samples.

## Introduction

Sumatriptan (SUM; IUPAC name: 1-[3-(2-Dimethylaminoethyl)-1H-indol-5-yl]-N-methyl-methanesulfonamide) is the first clinically available tryptamine-based drug which is commonly used to treat acute migraine attacks^1^. The compound, classified as a serotonin agonist, targets the 5-HT1D and 5-HT1B receptors, effectively mitigating vascular inflammation associated with migraine^2^. It is found that after oral administration of a sumatriptan 50 mg tablet, the maximum concentration in human blood plasma (*C*_max_) is 33.21 ng/mL (0.11 μM) after *t*_max_ of 1.13 h and the elimination half-life (*t*_1/2_) is 2.96 h^3^. It is worth noting that high doses of SUM (200 mg day^−1^) may lead to sulfhemoglobinemia, the situation in which the addition of sulfur into the hemoglobin molecules occurs and the blood color changes from red to greenish-black^4^.

Due to the physiological importance of SUM, extensive efforts have been devoted to determining this compound in biological samples. Among the diverse analytical methods available, liquid chromatography has been advised as a typical technique, and diverse liquid chromatographic techniques such as HPLC^5–7^, UPLC^8^, and HPTLC^9^ have been developed for its measurement. Additionally, alternative methods including micellar electrokinetic chromatography^10^, conductometry^11^, UV–Vis spectrophotometry^12,13^, capillary electrophoresis^14^, and fluorescence spectroscopy^15^ have been explored in the literature for determination of SUM. Despite notable sensitivity in some cases, these methods often suffer from tedious and time-consuming procedures, and high costs, which require specific standards, complexing agents, or even toxic solvents for analysis. Consequently, there is a compelling need for the development of sensitive, cost-effective, rapid, and straightforward approaches for the determination of SUM. These characteristics find fulfillment in electrochemical sensors, as previous studies have demonstrated the electrochemical activity of SUM^16^. Accordingly, some efforts have been invested in development of electrochemical sensing platforms for the sensitive determination of SUM.

Following the pioneering work by Sagar et al.^16^, which employed a glassy carbon electrode (GCE), subsequent researchers have used chemically modified electrodes to facilitate electron transfer between the electrode surface and SUM as the analyte, thereby enhancing detection sensitivity. Notable examples include the application of pyrolytic graphite electrode improved by multi wall carbon nanotubes (MWCNTs) decorated with silver nanoparticles^17^, carbon-paste electrode modified with a Cobalt Schiff-base coordination complex and MWCNTs^18^, ion-selective electrode incorporating dioctyl phthalate within carboxylated polyvinyl chloride matrix^19^, and carbon paste electrode modified by ultrasonic-electrodeposited Pt nanoparticles on ZrO_2_ nanoparticles^20^. Furthermore, various modifications of GCE (such as bilayer structures consisting of MWCNTs/polypyrrole^21^, Cu nanoparticles/poly-melamine^22^, and self-assembled MXene-MWCNT-chitosan^23^) have also been explored. In all cases, modified electrodes have consistently outperformed their unmodified counterparts in SUM determination.

Pencil graphite, one of the most extensively used electrode materials, is known for its significantly low detection limits, low background signal, high sensitivity and reproducibility, modifiable electroactive surface area, cost-effectiveness, and disposability. Therefore, the pencil graphite electrode (PGE) has attained significant consideration in recent years as a valuable alternative for traditional expensive electrodes^24,25^. The literature survey underscores that the electrocatalytic properties of PGE can be improved by modifying the electrode with suitable electrocatalyst/electron mediators^26–30^, as well as electrochemical pretreatment^31,32^, and delamination^33^. Notably, numerous efficient electrochemical sensor platforms for the determination of various compounds in biological samples have been presented on the base of PGE and electrochemically treated PGE^34^.

In the realm of analytical techniques, the development of electrochemical electrodes has shown remarkable growth due to their versatility and affordability. Therefore, electrodes with new nanocomposite interfaces have emerged^35–42^. On the other hand, metal–organic frameworks (MOFs), which are porous coordination polymers consisting of metal-based nodes and multitopic organic ligands, have been applied as electrode modifier materials. Due to their porous nature, ease of tailoring, ease of modification with various organic dyes and nanostructures, high mechanical stability, conductivity, and catalytic properties, MOFs have received significant attention as electroactive interfaces for electrochemical sensors^43^. Electrochemical treatment of PGE in a mixture of melamine and ammonium sulfate has been reported for in-situ synthesis of sulfur and nitrogen co-doped graphene nanocomposite on PGE (SNDGr/PGE). The obtained SNDGr/PGE was utilized as a support for the electrodeposition of Cu-MOF by applying a suitable cathodic potential^44^. The electrodeposition technique was also used in the fabrication of a binder-free cobalt-doped Ni-MOF film on GCE for direct sensing of levofloxacin^45^. Also, core–shell Fe_3_O_4_@ZIF‑8 nanoparticles (ZIF-8 = Zeolitic imidazolate framework-8) immobilized on screen printed graphite electrode has been utilized for voltammetric sensing of sumatriptan in the presence of naproxen in aqueous solutions^46^.

In numerous studies, MOFs have been immobilized on the electrode surface by the casting method to fabricate the chemically modified electrodes^47^. Moreover, in some cases, supporting materials have also been added^48–50^. However, we suppose that by electrochemical treatment of PGE, MOF nanoparticles could efficiently be immobilized on the electrode surface. In this method, the electron transfer rate enhances while the supporting material is not required^51^.

Here, a simple, cost-effective, and straightforward electrochemical sensor is introduced for the determination of SUM in biological fluids. {[Zn_4_(1,4-bdc)_4_(bpda)_4_]0.5DMF.3H_2_O}_n_ (Zn(II)-MOF, where 1,4-bdc = 1,4-benzene dicarboxylate, bpda = N,N′-bis(pyridine-4-yl)-1,4-benzenedicarboxamide) is a two-fold interpenetrated amide-containing MOF with open-ended channels^52,53^. The compound has previously been investigated for CO_2_ adsorption within its open-ended channels. However, more recently, it has been utilized through a simple casting method to fabricate a modified screen-printed carbon electrode (SPCE) which applied for the measurement of Fentanyl^47^. In this work, hydrothermally synthesized Zn(II)-MOF (as a modifier) was drop-casted on the surface of an electrochemically delaminated PGE (EDPGE). Electro-catalyzed oxidation of SUM on the surface of Zn(II)-MOF modified EDPGE was investigated for determination of the compound in biological matrices. The fabricated sensor exhibits a linear response in the concentration range of 0.99 to 9.52 µM for SUM solutions in human blood serum. To the best of our knowledge, the introduced Zn(II)-MOF/EDPGE sensor has not been employed for the determination of SUM previously.

## Experimental

### Reagents and materials

All reagents and solvents used, except for sumatriptan, were of analytical grade and were purchased from Merck and Sigma Aldrich Companies (Germany) and used as received without any further purification. Zinc chloride (ZnCl_2_, 99.9%), dimethylformamide* (*DMF, anhydrous, 99.8%), 2-amino-1,4-benzene dicarboxylic acid (NH_2_BDC, 2-aminoterephthalic acid, 99%**),** sodium hydroxide (NaOH, 99.9%), potassium chloride (KCl 99.9%), potassium hexacyanoferrate (III) (K_3_[Fe(CN)_6_] , 99.9%) were used through the study. N,N′-Bis(4-pyridinyl)-1,4-benzenedicarboxamide (BPDA, Purity: > 99%) was synthesized according to the previously reported method^54^. The raw material of sumatriptan (brand name: Imitrex) with a purity of 99.9% was obtained from Tehran-Shimi Company (Iran). A stock solution (2 mM) was prepared by dissolving 29.54 mg of SUM in 50 mL of deionized (DI) water. A 0.1 M KCl solution containing 1 mM [Fe(CN)_6_]^4−/3−^ was applied for the electrochemical impedance spectroscopy (EIS) studies. The respective Materials Safety Data Sheets (MSDSs) are given in supplementary information.

### Instruments

For electrochemical experiments, a PGSTAT-12 potentiostat/galvanostat device (ECO Chemie, Netherlands) equipped with GPES software was used. In addition, electrochemical impedance spectroscopy (EIS) was performed by Ivium potentiostat/galvanostat (CompactStat.h, Ivium Technologies BV) linked to Ivium software. For electrochemical research work, a pencil core with diameter of two mm and HB hardness (German Routing Company) was used. The modified pencil graphite was used as the working electrode. An Ag/AgCl and a Pt wire were used as reference and counter electrodes, respectively. All electrochemical measurements were performed at room temperature.

The synthesized materials and the electrode surface were characterized using FTIR, SEM, XRD, TGA, and EDX techniques. The infrared spectrum (FTIR) was recorded, in the range of 4000–400 cm^−1^, using KBr pellets on a Win-Bomem spectrometer (Version 3.04 Galatic Industries Corporation and Thermo Nicolet Nexus 670). To identify the synthesized compounds, Powder XRD (PXRD) data were collected at 297 K on Bruker D8 Advance equipped with CuK_α_ radiation (λ = 0.15406 nm). Scanning electron microscopy (SEM) images and EDX analyses were recorded using a ProX model SEM (Netherlands) with a gold coating thickness of up to several angstroms.

### Synthesis of the Zn(II)-MOF

**Figure 1:**
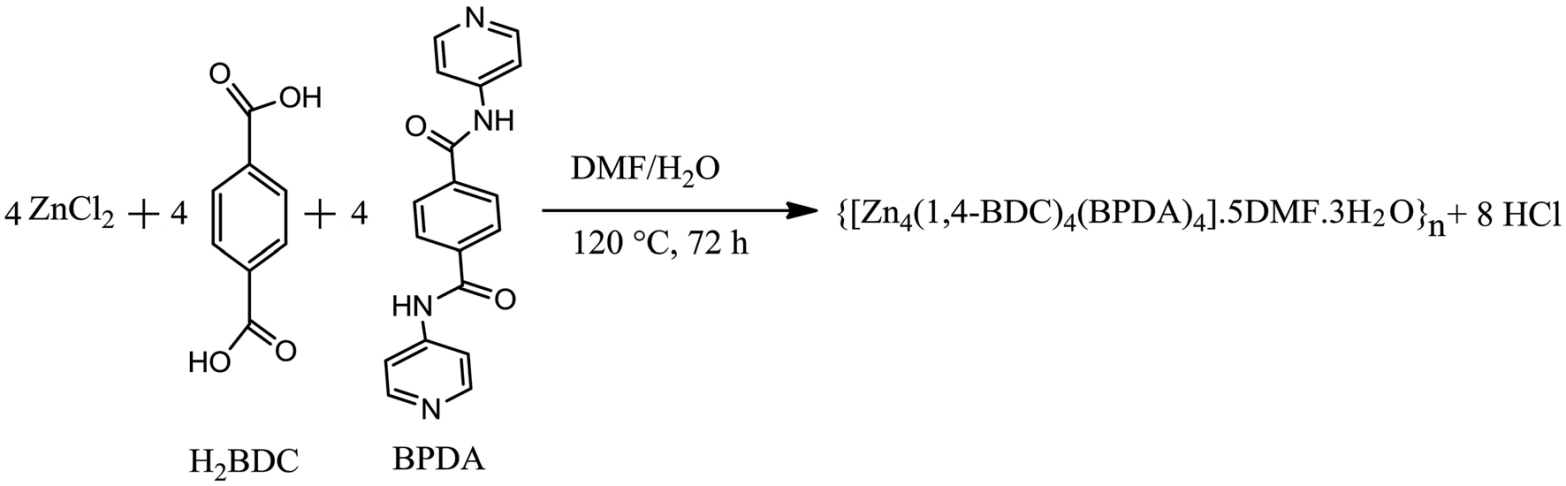
The balanced reaction equation of the hydrothermal synthesis reaction.

**Figure 2:**
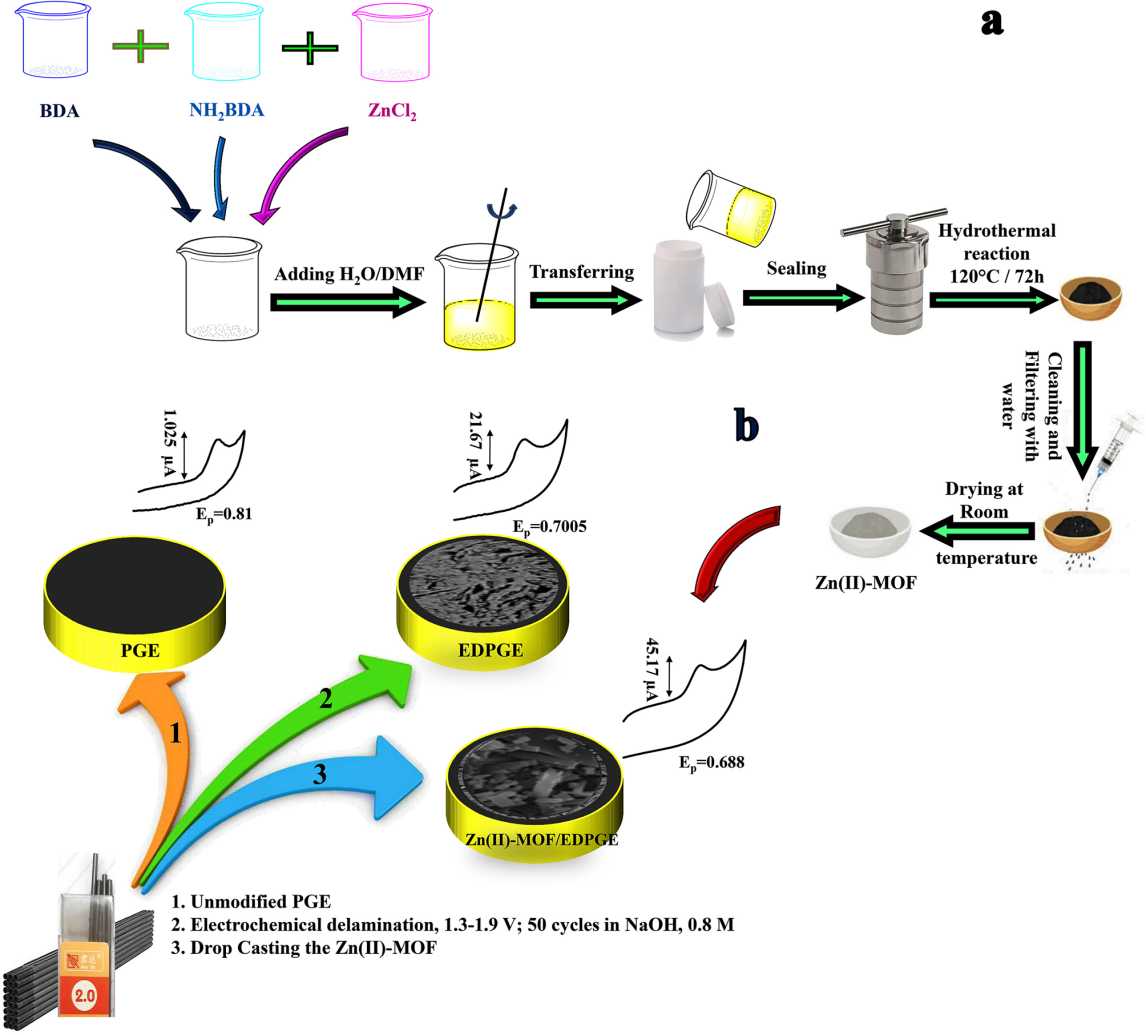
Schematic illustration of the synthesis of Zinc(II)-MOF (**a**) and Preparation of Zn(II)-MOF/EDPGE (**b**).

Zn(II)-MOF was synthesized according to the previous reports^47,52^. Figures 1 and 2a schematically shows the synthesis steps. Bis-(4-pyridinyl)-1,4-benzene dicarboxamide (BPDA, 0.15 mmol, 47.8 mg), 2-amino-1,4-benzene dicarboxylic acid (BDC NH_2_, 0.15 mmol, 47.8 mg) and ZnCl_2_ (0.15 mmol, 20.5 mg) were dissolved in 0.5 mL of water and 7 mL of DMF. The resulting solution was then transferred to a Teflon-lined autoclave and heated at 120 °C for 72 h. After cooling, Zn(II)-MOF was precipitated as a powder which was filtered, washed with water and ethanol, and left to air-dry at room temperature. IR (KBr): ν = 3466 (m), 3366 (m), 3085 (w), 1677 (s), 1597 (vs), 1569 (s), 1503 (s), 1430 (s), 1379 (s), 1332 (s), 1298 (s), 1261 (m), 1211 (m), 1119 (m), 1017 (m), 890 (m), 834 (m), 712 (m), 603 (m), 538 (m) cm^−1^.

### Preparation of EDPGE and Zn(II)-MOF/EDPGE

A schematic representation of the preparation of the modified electrode is given in Fig. 2b. To create a cost-effective sensor, a pencil lead was used as the base of the electrode. The electrode was prepared in a two-step procedure. First, the PGE was delaminated through an electrochemical method. To establish an electric current, a copper wire was attached to the end of the pencil lead, and the entire electrode body was insulated with Teflon tape. Subsequently, the electrode surface was polished using soft sandpaper and thoroughly rinsed with distilled water. Then, the pencil graphite electrode was immersed in an electrochemical cell containing 0.8 M sodium hydroxide solution. The electrode surface was activated using the cyclic voltammetry (CV) technique and applying the potential in the range of + 1.3 to + 1.9 V. The electrochemically delaminated pencil graphite electrode (EDPGE) was achieved after 50 cycles.

In the next step, to prepare the Zn(II)-MOF/EDPGE, Zn(II)-MOF was deposited onto the EDPGE surface. To prepare a uniform dispersion of the Zn(II)-MOF nanoparticles in water, 10 mg of the synthesized compound was added to 5 mL of distilled water and subjected to ultrasound waves for 1 h. 5 μL of the prepared water-dispersed Zn(II)-MOF nanoparticles was drop-casted on the prepared EDPGE. The electrode was left to air-dry for 24 h before use.

### Preparation of human blood serum for analysis

Human blood was obtained from the Pasteur Laboratory (Khoy, Iran) and stored in a refrigerator for 24 h to allow for clotting before use. To strip and remove proteins, 1.5 mL of methanol was added to 2 mL of blood serum. The resulting mixture was centrifuged for 10 min at 3000 rpm to separate the clear solution. Subsequently, the solution was filtered through a 0.2 µm syringe filter. The filtered solution was then brought to a volume of 10 mL with phosphate buffer (pH = 7). The resulting matrix was used for the analysis of SUM using spike studies^55^.

### Statement

All experiments and methods conducted throughout this study strictly adhered to relevant guidelines and regulations. In addition, it is confirmed that all participants involved in this research signed informed consent. Furthermore, it is declared that all research and methods involving human subjects were carried out in accordance with the ethical standards and regulations of the Ethical Committee of the Urmia University of Medical Sciences, Urmia, Iran, under permission number 3-235686, granted on July 12th, 2021. Before their participation, the volunteers received comprehensive information about the experimental aspects of the study. They were assured of anonymity in the experimental process and also the publication of results exclusively for scientific purposes.

## Results and discussion

### Characterization of Zn(II)-MOF

#### Morphology analysis

**Figure 3:**
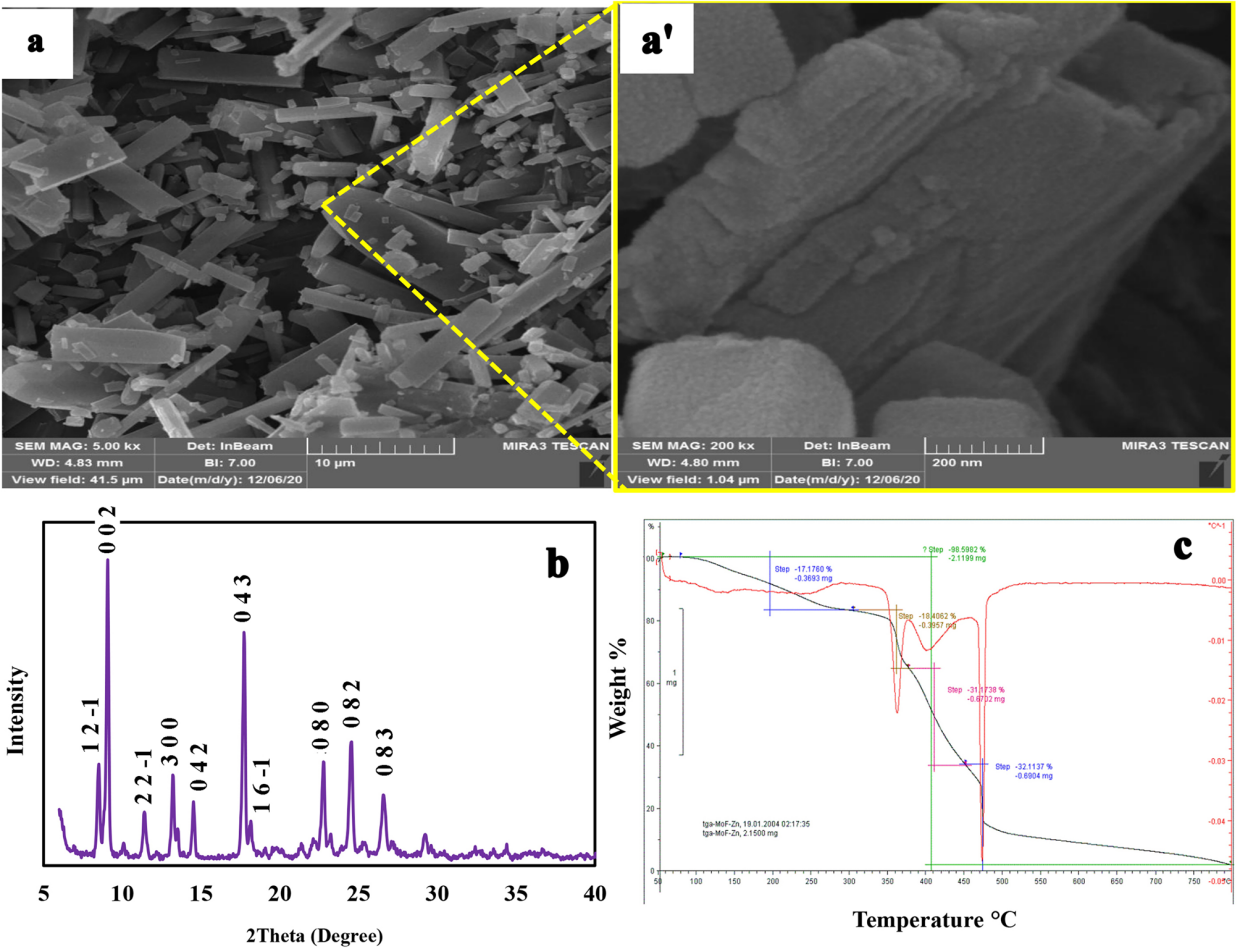
SEM images (**a**, 5000 × and **aʹ**, 200,000 ×), XRD pattern (**b**), and TGA **(c)** for the synthesized Zn(II)-MOF.

The morphology of the hydrothermally synthesized Zn(II)-MOF was examined by scanning electron microscopy (SEM). As shown in Figs. 3a and **aʹ**, under hydrothermal conditions, micrometer-sized plate-like crystals were formed by aggregation of Zn(II)-MOF nanocrystals.

#### FTIR spectrum

The FTIR spectrum of the synthesized Zn(II)-MOF is shown in Fig. S1 in the supplementary information. The observed bands in the measured spectrum of the compound are in accord with the previously reported values^52^. The lattice water ν (O–H) stretching vibration is observed as a broad medium band at 3466 cm^−1^. The ν (N–H) stretching band is found at 3366 cm^−1^. The band at 3064 cm^−1^ is due to the aromatic ν (C–H) stretching vibration. The amide N–H bending vibration is observed as a strong band at 1677 cm^−1^. The very strong band at 1597 cm^−1^ and the strong band at 1569 cm^−1^ are attributable, respectively, to ν (C=N) and ν (C=O) stretching vibrations. The carboxylate COO bending vibrations observed as a strong band at 1332 cm^−1^. Also, the C–O stretching vibration is observed at 1119 cm^−1^.

#### PXRD analysis

The measured PXRD pattern of the synthesized Zn(II)-MOF (Fig. 3b) is comparable with previous reports^47,52,56^, and confirms successful synthesis of the desired compound. The measured PXRD data was analysed using Reflux module of Materials studio (v8, Accelrys)^57^. Crystal structure (cell parameters, atom positions, and temperature factors) determined by single crystal XRD analysis^52^ was used for Rietveld refinement^58,59^ of the crystal structure against experimental PXRD data. In the previously reported single crystal structure^52^, the hydrogen atoms were appropriately adjusted on C and N atoms in calculated positions Materials studio^57^. The water hydrogen atoms were ignored in the refinement. Fractional coordinates of atoms were fixed and the temperature factors, calculated by single crystal x-ray diffraction, were not refined. Only the measure PXRD data in the range of 2θ = 8.00–45.00° were included in the refinement. Cell parameters were refined and the final refinement yielded the residuals Rp = 13.95% and Rwp = 17.99%. For the monoclinic unit cell (space group: *P*2_1_/*c*), the Rietveld refined lattice parameters [single crystal x-ray results^52^] are: *a* = 20.21525(± 0.00685) Å [20.1818(3) Å], *b* = 31.33971(± 0.00598) Å [30.2868(5) Å], *c* = 19.62813(± 0.00784) Å [19.7451(3) Å], and *β* = 89.14761(± 0.06117)° [90.6880(6)°]. Figure S2 in the supplementary information shows the final Rietveld plot for the refinement of Zn(II)-MOF. Also, the crystal packing of Zn(II)-MOF is given in Fig. S3 in the supplementary information. A Reflex summary report for the final Rietveld refinement of Zn-MOF and the crystal structure of the compound (in cif file format) are provided as the supplementary material. In the measured PXRD pattern, the reflection peaks observed at 2θ (indexed as) = 8.52° (1 2 −1), 9.08° (0 0 2), 11.4° (2 2 −1), 13.2° (3 0 0), 14.52° (0 4 2), 17.72° (0 4 3), 18.16° (1 6 −1), 22.76° (0 8 0), 24.56° (0 8 2) and 26.56° (0 8 3).

#### TGA

The thermal stability of the synthesized MOF was evaluated using TGA technique (Fig. 3c). In an inert atmosphere, the sample undergoes weight loss in four steps, which is a typical behavior observed for a mixed ligand containing MOF^60^. In the first step, between 100 and 300 °C, lattice water and DMF molecules leave the porous network of Zn(II)-MOF (weight loss of 17.17%). In the second step, a weight loss of 18.4% was observed at 300–370 °C which is related to the removal of BDC NH_2_. In the third step, the weight loss of 31.17% at 410–460 °C could be attributed to the elimination of BPDA and BDC NH_2_. Finally, between 470 and 700 °C, a weight loss of 32.11% occurs corresponding to the decomposition of BPDA. Since the test was performed in the absence of oxygen, the final product is zinc which is in accord with the theoretical value of 2.69%^53^.

### Characterization of the sensing interface

**Figure 4:**
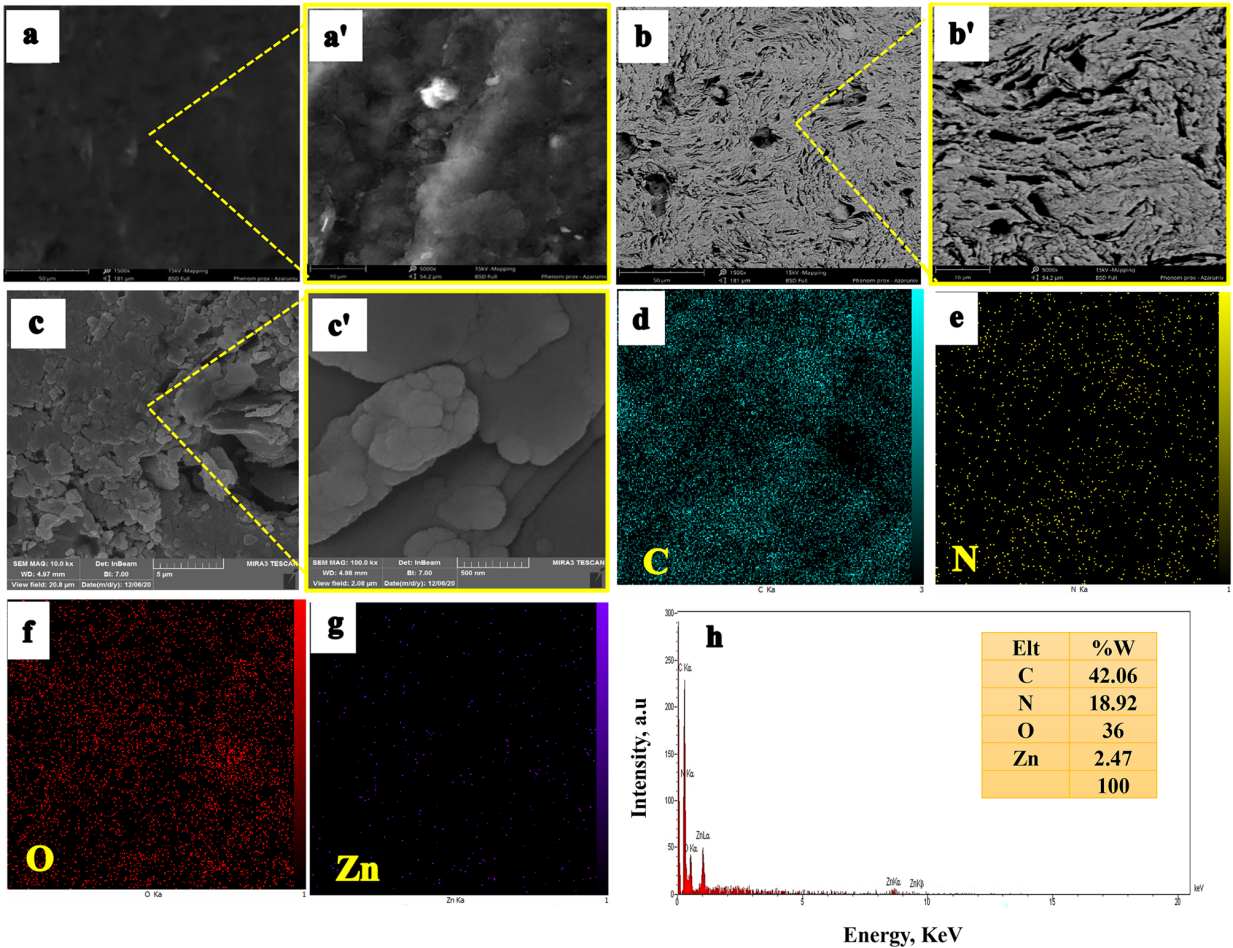
SEM images of the surface of pencil graphite electrode (PGE) (**a**, **aʹ**), the electrochemically delaminated electrode (EDPGE) (**b**, **bʹ**) and the final modified electrode (Zn(II)-MOF/EDPGE) (**c**, **cʹ**); EDS mapping of elements C (**d**), N (**e**), O (**f**) and Zn (**g**) and EDX analysis of Zn(II)-MOF/EDPGE (**h**).

The surface structure and morphologies of the unmodified/modified electrodes were surveyed by scanning electron microscopy (SEM). As shown in Fig. 4a, at the micrometer scale, the bare PGE has a relatively smooth cloud-like surface with no porosity. In comparison, graphite sheet fractures are visible for EDPGE (Fig. 4b), indicating that the electrochemical delamination has been performed successfully. The observed surface feature is consistent with previous studies^33,44^. Figure 4c illustrates that the EDPGE surface is effectively modified by the synthesized Zn(II)-MOF. The compound is well dispersed on the EDPGE surface and especially within its porous layers. Zn(II)-MOF nanoparticles are deposited throughout the surface of the electrode as porous lumps.

The energy dispersive spectroscopy (EDS) analysis was used to identify the constituent elements of the sample and to determine their relative weight percentages. Figure 4d–g depict EDS mapping analysis results, indicating the even distribution of the constituents’ elements including Zn, O, N, and C on the surface of Zn(II)-MOF/EDPGE. The EDX spectrum for the Zn(II)-MOF/EDPGE surface is shown in Fig. 4h. The presence of Zn, O, N, and C elements are confirmed and surface elemental analysis results are: C 42.06%, N 18.92%, O 36%, and Zn 2.47%. The Zn content is in good agreement with the value obtained from the TGA (2.69%).

**Figure 5:**
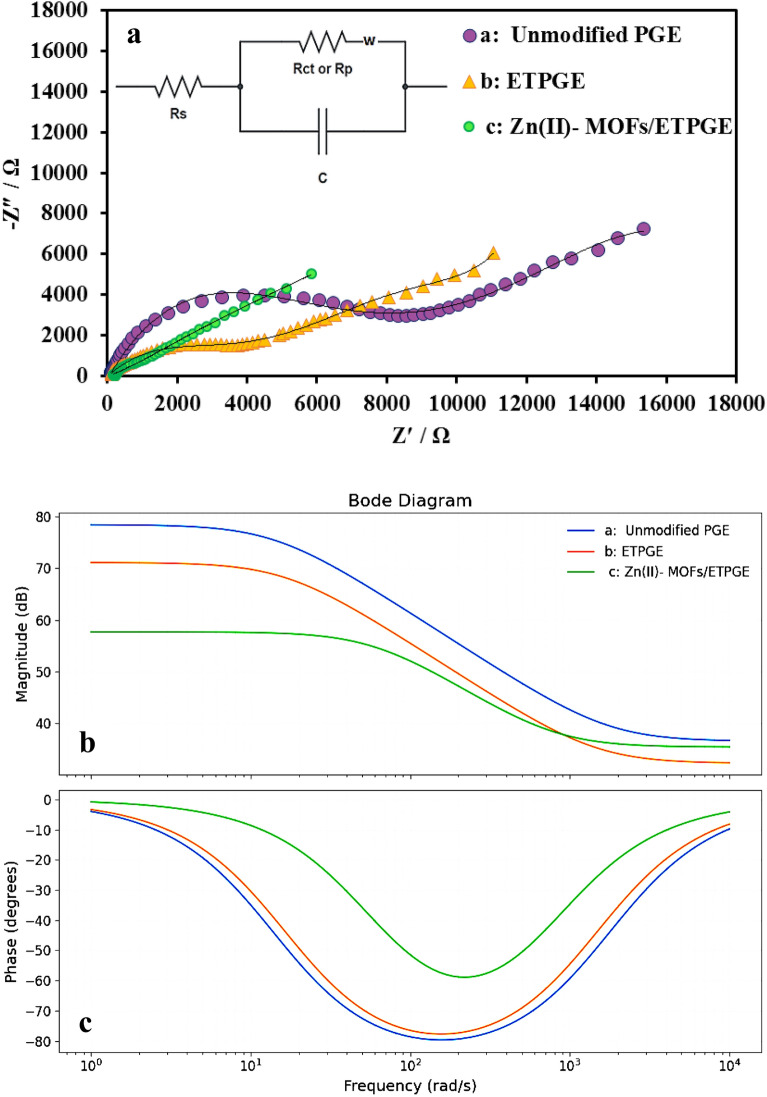
Nyquist plots of PGE, EDPGE, and Zn(II)-MOF/EDPGE measured in 0.1 M KCl solution containing 1 mM [Fe(CN)_6_]^−3/−4^ (inset: the equivalent Randles circuit model; the fitted Nyquist curves are given as solid black lines) (**a**) The respective bode plots depicting the magnitude changes vs frequency (**b**) and phase angle changes vs frequency (**c**).

Electrochemical Impedance Spectroscopy (EIS) is a very sensitive technique for the analysis of changes in electrode surface properties resulting from modifications. Also, it is useful to clarify the mass and charge-transfer, as well as diffusion processes at the electrode surface^61^. Figure 5a shows the Nyquist plots for PGE, EDPGE, and Zn(II)-MOF/EDPGE measured using Fe(CN)_6_^3−/4−^(1 mM)/potassium chloride (0.1 M) solution.

According to the obtained equivalent circuit (Fig. 5a), the following equation governs the behavior of impedance in the complex plane:

$$ {\text{Z}} = {\text{R}}_{{\text{s}}} + \frac{{{\text{R}}_{{\text{p}}} }}{{1 + {\text{j}}\omega {\text{R}}_{{\text{p}}} {\text{C}}}} = {\text{R}}_{{\text{s}}} + \frac{{{\text{R}}_{{\text{p}}} }}{{1 + \left( {\omega {\text{R}}_{{\text{p}}} {\text{C}}} \right)^{2} }} - {\text{j}}\frac{{\omega {\text{R}}_{{\text{p}}}^{2} {\text{C}}}}{{1 + \left( {\omega {\text{R}}_{{\text{p}}} {\text{C}}} \right)^{2} }} $$

$$ {\text{R}} = {\text{R}}_{{\text{s}}} + \frac{{{\text{R}}_{{\text{p}}} }}{{1 + \left( {{\omega R}_{{\text{p}}} {\text{C}}} \right)^{2} }}\quad {\text{X}} = { }\frac{{ - {\omega R}_{{\text{p}}}^{2} {\text{C}}}}{{1 + \left( {{\omega R}_{{\text{p}}} {\text{C}}} \right)^{2} }} $$

By removing the frequency parameter in the real and imaginary components, the following equation is obtained which governs the impedance behavior in the complex plane.

$$ \left( {{\text{R}} - {\text{R}}_{{\text{s}}} - \frac{{{\text{R}}_{{\text{p}}} }}{2}} \right)^{2} + {\text{X}}^{2} = \left( {\frac{{{\text{R}}_{{\text{p}}} }}{2}} \right)^{2} $$

Since $X<0$, this equation describes a half-circle in the lower complex plane with the following center and radius.

$$ {\text{Center}}:\;\;\left( {{\text{R}}_{{\text{s}}} + {\text{ R}}_{{\text{p}}} /{2},0} \right)\;\;{\text{Radius}}:\;\;\frac{{{\text{R}}_{{\text{p}}} }}{2} $$

The calculations were done as follows:

$$ \left\{ \begin{gathered} {\text{a:}}\;\left\{ {\begin{array}{*{20}c} {\begin{array}{*{20}c} {{\text{R}}_{{\text{s}}} + \frac{{{\text{R}}_{{\text{p}}} }}{2} = \frac{8313 + 67.45}{2}} \\ {\frac{{{\text{R}}_{{\text{p}}} }}{2} = 8313 - \frac{8313 + 67.45}{2}} \\ \end{array} \to {\text{R}}_{{\text{p}}} = 8245.55 , {\text{R}}_{{\text{s}}} = 67.45} \\ { \to \omega = \frac{1}{{{\text{R}}_{{\text{p}}} {\text{C}}}} in f = 2.25 Hz \to C = 8.578 \mu F} \\ \end{array} } \right. \hfill \\ {\text{b}}:\;\left\{ {\begin{array}{*{20}c} {\begin{array}{*{20}c} {{\text{R}}_{{\text{s}}} + \frac{{{\text{R}}_{{\text{p}}} }}{2} = \frac{3583 + 41.26}{2}} \\ {\frac{{{\text{R}}_{{\text{p}}} }}{2} = 3583 - \frac{3583 + 41.26}{2}} \\ \end{array} \to {\text{R}}_{{\text{p}}} = 3541.74 , {\text{R}}_{{\text{s}}} = 41.26} \\ { \to \omega = \frac{1}{{{\text{R}}_{{\text{p}}} {\text{C}}}} in f = 2.67 Hz \to C = 16.83 \mu F} \\ \end{array} } \right. \hfill \\ {\text{c}}:\;\left\{ {\begin{array}{*{20}c} {\begin{array}{*{20}c} {{\text{R}}_{{\text{s}}} + \frac{{{\text{R}}_{{\text{p}}} }}{2} = \frac{764.5 + 59.12}{2}} \\ {\frac{{{\text{R}}_{{\text{p}}} }}{2} = 764.5 - \frac{764.5 + 59.12}{2}} \\ \end{array} \to {\text{R}}_{{\text{p}}} = 705.38 , {\text{R}}_{{\text{s}}} = 59.12} \\ { \to \omega = \frac{1}{{{\text{R}}_{{\text{p}}} {\text{C}}}} in f = 0.967 Hz \to C = 233.33 \mu F} \\ \end{array} } \right. \hfill \\ \end{gathered} \right. $$

The equivalent Randles circuit model is depicted in the inset of Fig. 5a, which schematically describes the electrochemical processes at the interface surface. The fitting parameters for the studied electrodes are provided in Table 1. The R_s_, R_ct_ or R_p_, W and C, respectively, are voltage source resistance, charge transfer resistance, parallel resistance, Warburg impedance and the double-layer capacitance (C_dl_)^62^.

**Table 1:** The fitting parameters for the studied electrodes.

Electrode	R_s_ (Ω)	R_p_ or R_ct_ (Ω)	C (F)
PGE	67.45	8245.55	8.578 μ
EDPGE	41.26	3541.74	16.83 μ
Zn(II)-MOF/EDPGE	59.12	705.38	233.33 μ

The Nyquist plot of the PGE consists of a semicircular section with a large diameter followed by a straight line. Accordingly, the charge transfer resistance (R_ct_) value for bare PGE was estimated to be 8245.55 Ω. In comparison, the electron transfer rate for EDPGE is enhanced and the R_ct_ was reduced to 3541.74 Ω. On the other hand, by depositing Zn(II)-MOF on the active sites of the EDPGE, the electron transfer resistance decreases considerably to a value of 705.38 Ω. The EIS measurements indicate that step-by-step modification of PGE results in the enhanced charge transduction rate, accelerated electrode kinetics, and increased conductivity at the electrode interface.

A Bode plot is the second main format for graphical representation of the impedance data and is used to identify charge-transfer processes^63^. It is a powerful tool to study the frequency response of a system. By changing the frequency, the reactance occurred between the two interface plates is investigated to understand the changes in the apparent impedance. Figures 5b and c represents the bode plots for PGE, EDPGE, and Zn(II)-MOF/EDPGE. Bode plots in the form of phase angle (θ) versus frequency (Fig. 5c) are characterized by a well-defined peak corresponding to one distinct relaxation process^64^. Here, Bode plots clearly confirm the Nyquist plot findings.

Figure S4A in the supplementary information gives the chronocoulograms for bare PGE, EDPGE, and Zn(II)-MOF/EDPGE, recorded in Fe(CN)_6_^3−/4−^ (1 mM) solution. The electrochemical active surface area of all electrodes was calculated using the Cottrell equation^65^:

$$ Q = 2nFACD^{1/2} t^{1/2} \pi^{ - 1/2} + \, Q_{dl} + \, Q_{ads} $$

where Q_dl_ and Q_ads_ are, respectively, the charge of double-layer and adsorbed species, n is the number of electrons exchanged in the reaction, A is the surface area of the electrode (cm^2^), C is the solution concentration (mol cm^−3^), and D is the diffusion coefficient (cm^2^ s^−1^). The Q *vs.* t^1/2^ graph is shown in Fig. S4B in the supplementary information. The slope values calculated for bare PGE, EDPGE, and Zn(II)-MOF/EDPGE are 4.79, 9.76, and 19.60, respectively. Also, the calculated electrode surface area (A) values are 0.016 cm^2^, 0.0322 cm^2^, and 0.065 cm^2^ for bare PGE, EDPGE, and Zn(II)-MOF/EDPGE. Accordingly, the surface area of EDPGE and Zn(II)-MOF/EDPGE are approximately two and four times higher than that of bare PGE. Such a property is a prerequisite for the development of highly sensitive sensors.

### Electrochemical behavior of sumatriptan on Zn(II)-MOF/EDPGE sensor

**Figure 6:**
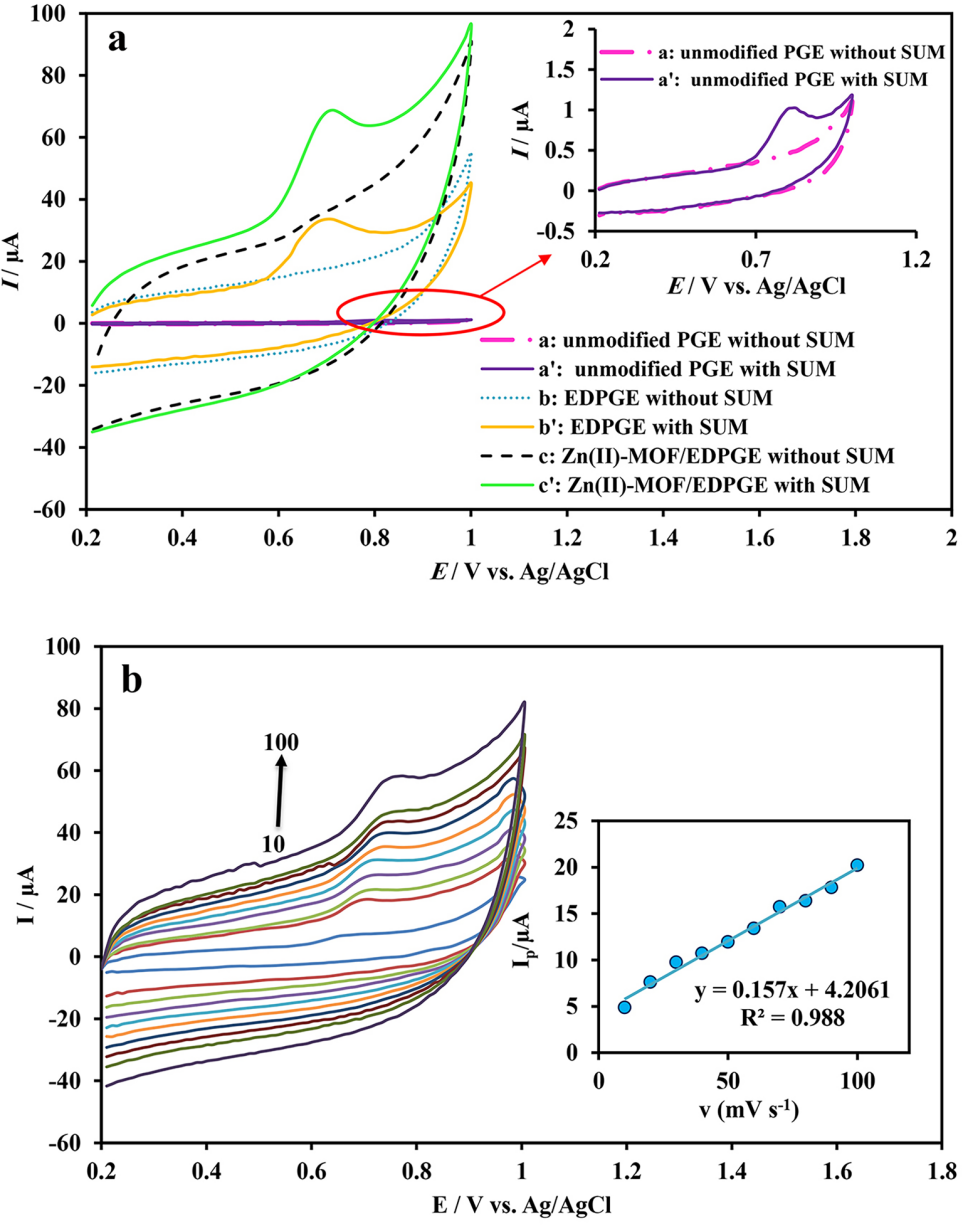
Cyclic voltammograms for bare PGE, EDPGE, and Zn(II)-MOF/EDPGE measured in 0.1 M phosphate buffer (pH = 7) in the absence and presence of SUM (90.9 μM) (**a**) and cyclic voltammograms in 0.1 M phosphate buffer solution in the presence of SUM (50.7 μM) at different scan rates (10–100 mV/s) (inset: Changes in the anodic peak current *vs.* the scan rate) (**b**).

Cyclic voltammetry has been widely used to study electron transfer processes, the effect of chemical reactions on electrode surface electrochemical processes, as well as to evaluate stability properties and electrocatalytic activities^66^. To assess the electrocatalytic activity of the synthesized Zn(II)-MOF, acting as a surface modifier of EDPGE in the electro-oxidation of SUM, cyclic voltammograms of the bare PGE, EDPGE, and Zn(II)-MOF/EDPGE (in 0.1 M phosphate buffer at pH 7) were measured in the absence and presence of SUM (90.9 μM) (see Fig. 6a). In the absence of SUM, no electrochemical signal was observed for the bare PGE. However, in the presence of SUM, a weak oxidation signal at 0.81 V with a current of 1.025 µA was detected (Fig. 6a, curve a). In comparison, for the EDPGE, the background current was significantly increased due to the augmentation of the electrode's active surface (Fig. 6a, curve b).

By electrochemical pretreatment process, various oxygen-containing functional groups are formed on the surface^44^. Moreover, the process leads to the formation of graphite sheet fractures due to the penetration of OH^−^ groups and solvent molecules into the graphite layers^33^. The expansion of graphite sheet fractures exposes new layers to oxidation, resulting in an excellent surface for the deposition of the synthesized MOF. By adding the analyte to the buffer, the electro-oxidation signal increases, while the overvoltage is reduced due to the pretreatment of the electrode. Cyclic voltammograms of the Zn(II)-MOF/EDPGE in the absence and presence of SUM are illustrated in Fig. 6a, curve **c**. Due to the formation of active sites on the surface of Zn(II)-MOF/EDPGE, compared to the PGE and EDPGE, the resulting signal is significantly amplified. As a result, the current intensity is approximately 20 times greater than that observed for the bare PGE.

### Investigation of experimental parameters for SUM oxidation on Zn(II)-MOF/EDPGE surface

The amount of the electrode modifier compound and the accumulation time are the most important factors which affect the electrode performance. To optimize these parameters, 1, 3, 5, 10, and 15 μL of the prepared Zn(II)-MOF suspension (10 mg in 5 mL H_2_O) were pipetted and deposited on the EDPGE surface. The highest sensitivity of Zn(II)-MOF-modified EDPGE was achieved with 5 μL of the suspension. For this electrode, the anodic peak current reached its maximum value (see Fig. S5A in the supplementary information) may be due to the availability of the active microscopic surface for the adsorption and oxidation of SUM. By increasing the used suspension volume to 10, and 15 μL, the anodic peak current decreases. By using larger volumes of the modifier a thicker layer forms on the electrode surface resulting in the supersaturation of the adsorbed analyte molecules on the electrode surface. This phenomenon passives the electron transfer and electrode resistivity increases, which reduces the electrode response to the analyte^67^.

To investigate the effect of scanning rate, cyclic voltammograms of SUM were recorded using the working electrode at scan rates ranging between 10 and 100 mV/s. As shown in Fig. 6b, the anodic peak currents increase with higher scan rates, and the anodic peak potential shifts towards more positive potentials. Moreover, the oxidation and reduction peak currents in the measured cyclic voltammograms are found to be asymmetric. This observation suggests that the reaction is irreversible. In addition, it is found that the anodic current changes linearly with the scanning rate (see inset in Fig. 6b) indicating that the electrochemical reaction mechanism depends on the adsorption of electroactive species on the electrode surface^68^. The adsorption-based electro-oxidation of SUM has also been reported, previously, using MXene-MWCNT-chitosan/GCE^23^ and Pt-ZrO_2_ NPs/CPE^20^.

Since the electro-oxidation of SUM on Zn(II)-MOF/EDPGE surface follows an adsorption-depended mechanism, the accumulation time would be a crucial parameter that affects the electrochemical response of the sensor. The effect of accumulation time on sensor performance was investigated by differential pulse voltammetry (DPV) measurements for 5.82 µM SUM solutions (see Fig. S5B and C in the supplementary information). It is worth noting that differential pulse voltammetry (DPV) is a popular technique that exhibits more sensitive response rather than the CV. This is rooted in the fact that measuring the current for each pulse effectively minimizes the background current. Therefore, this technique is useful in the analysis of trace analytes.

Increasing the accumulation time up to 60 s led to an increase in the oxidation peak current of SUM on the surface of Zn(II)-MOF/EDPGE (Fig. S5B and C in the supplementary information). However, when the accumulation time exceeds 60 s the signal strength decreases due to the electrode surface saturation. As a result, an accumulation time of 60 s was chosen as the optimum duration for the subsequent studies.

**Figure 7:**
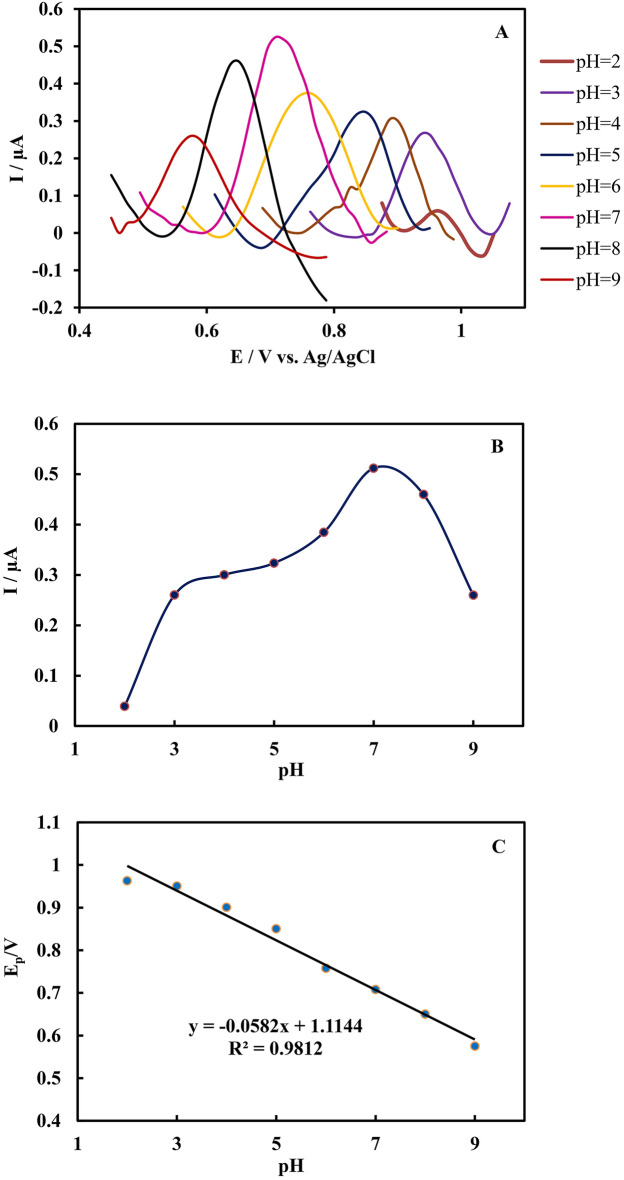
(**A**) The pH effect on the DPV measured for 8.61 μM SUM solution using Zn(II)-MOF/EDPGE (supporting electrolyte: phosphate buffer, 0.1 M), (**B**) Ipa changes with solution pH value, (**C**) The linear relationship between pH value and E_pa_.

The pH value of the supporting electrolyte is the other parameter which affects the electrochemical response of the Zn(II)-MOF/EDPGE. The effect of electrolyte pH on the oxidation of SUM was studied by DPV method in the pH values between 2.0 and 9.0 (Fig. 7). According to the DPV measurements, the best discrimination occurs at pH 7. As a result, the subsequent electrochemical measurements of SUM were carried out in pH 7. In addition, with increasing the solution pH, the SUM oxidation peak potential shifts toward less positive values. This foundation indicates that protons are involved in the oxidation of SUM. A linear relationship between the observed peak potential (Ep) and the pH value of the supporting electrolyte was found. The calculated linear regression equation is Ep (V) = 0.0582 pH + 1.1144 (R^2^ = 0.9812). The obtained slope value of 0.0582 V/pH is close to the theoretical value of 0.059 V/pH, indicating that an equal number of protons and electrons are involved in the respective electrode reaction.

### Analytical features of the developed Zn(II)-MOF/EDPGE in determination of SUM

**Figure 8:**
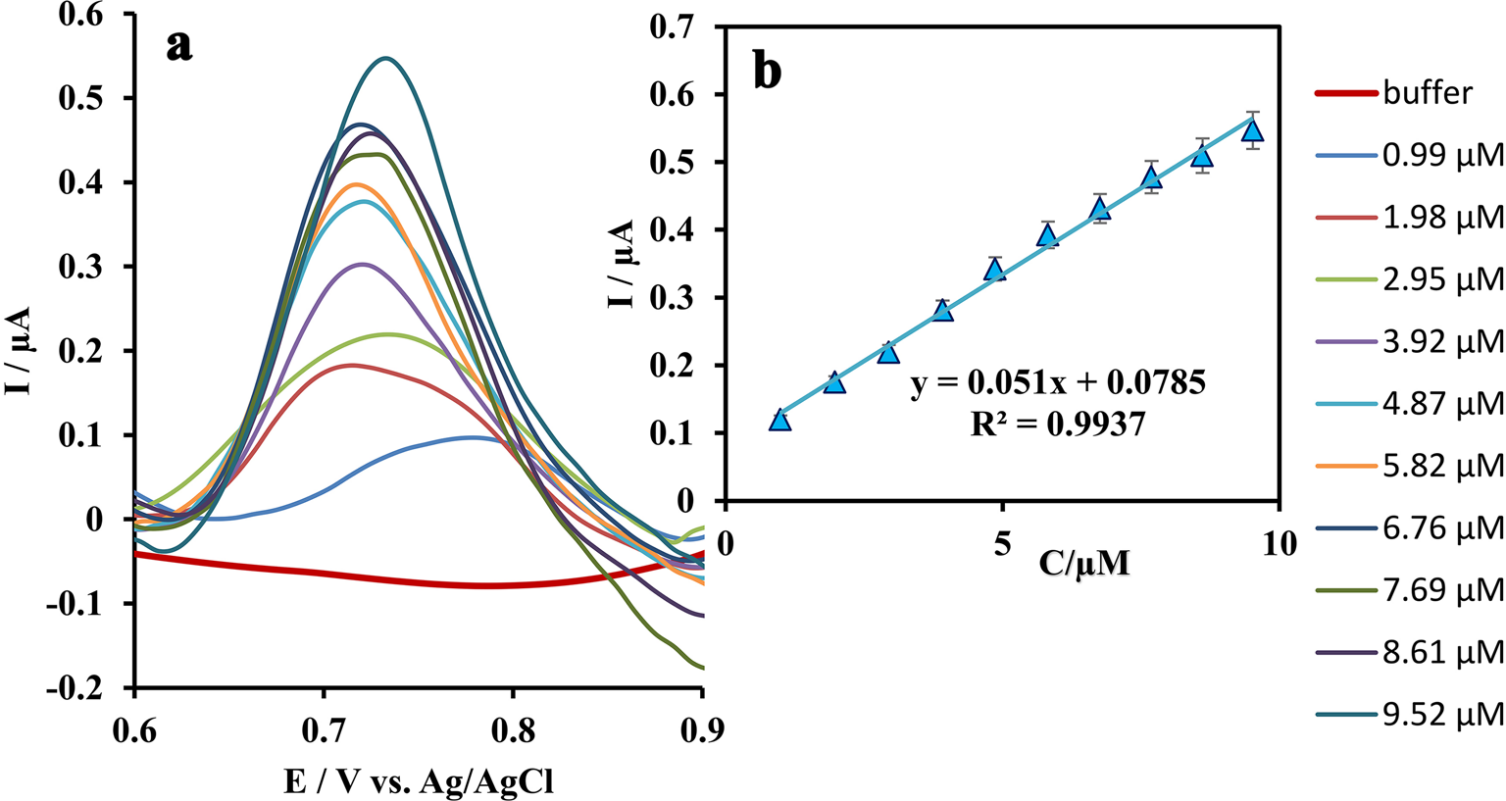
Pulse voltammograms recorded using Zn(II)-MOF/EDPGE in the absence (Blank) and in the presence of SUM (0.99 to 9.52 μM) in 0.1 M phosphate buffer solution (pulse amplitude: 50 mV, pulse width: 50 ms, and scan rate: 20 mV/s) (**a**) and calibration curve, plotting anodic electro-oxidation peak current *vs.* SUM concentration (each point shows the average value for three tests ± SD) (**b**).

At optimal conditions, the SUM concentration range for which the fabricated Zn(II)-MOF/EDPGE sensor responses linearly, was determined using DPV method in 0.1 M phosphate buffer solution (Fig. 8a). Pulse amplitude of 50 mV, pulse width of 50 ms, and scan rate of 20 mV/s were applied in the measurements. The calibration curve was obtained by plotting the anodic peak current *vs.* standard concentrations of SUM, and the regression equation was derived. It was found that when the SUM concentration (C_SUM_) changes between 0.99 and 9.52 μM, using Zn(II)-MOF/EDPGE, the respective oxidation peak current (i_p_) increases linearly (Fig. 8). The experimental detection limit for the described sensor was calculated to be 0.29 μM (LOD = 3S_b_/m; *S*_b_ = the standard deviations for the blank solution and m = the slope of the calibration graph). It should be noted that the DPV method allows investigating the concentrations until the modified electrode surface reaches saturation. After saturation, the active sites on the electrode can be easily reactivated by subjecting the electrode to cyclic voltammetry in the phosphate buffer.

Table 2 compares the fabricated Zn(II)-MOF/EDPGE sensor with some previously reported electrochemical SUM sensors^17,18,20–23^. Compared to the previous reports, the sensor introduced in this work has excellent features in terms of its straightforward design and cost-effectiveness. Also, it offers favorable characteristics considering its linear range and detection limit. The fabricated sensor benefits from easy and single-step activation of PGE followed by its modification through the casting of Zn(II)-MOF on its surface. As a result of electrochemical delamination, which introduces porosity to the PGE surface, the resulting EDPGE can be easily modified by drop-casting of Zn(II)-MOF. In contrast, the previously reported approaches suffer from multistep electrode preparation.

**Table 2:** Comparison of the different electrochemical sensors developed for determination of sumatriptan.

Electrode	Features	Method	Linear range (µM)	LOD (µM)	Ref
AgNPs-MWCNT/PyrGE	Multistep preparation, without treatment of PyrGE, Casting, Costly	CV	0.08–100	0.04	^17^
Cobalt Schiff-base-MWCNT/CPE	Multistep prepration, mixing, Handmade	DPV	1–1000	0.3	^18^
PPY-CNT/GCE	Multistep preparation, without treatment of GCE, Casting, Costly	DPV	0.02–10	0.006	^21^
Pt-ZrO_2_ NPs/CPE	Multistep prepration, mixing, Handmade	Amperometry	0.01–55	0.003	^20^
Cu NPs/poly-melamine/GCE	Multistep preparation, without treatment of GCE, Casting, Costly	DPV	0.58–6.5	0.025	^22^
MXene-MWCNT-chitosan/GCE	Multistep preparation, without treatment of GCE, Casting, Costly	DPV	0.0033–61	0.00042	^23^
Zn(II)-MOF/EDPGE	easy treatment of PGE, Casting, Cost-effectiveness of PGE	DPV	0.99–9.52	0.29	This work

### Interference study

Selectivity of Zn(II)-MOF/EDPGE sensor for SUM was evaluated by examining the interference effect of some species that could be present in biological samples (see Fig. S6 in the supplementary information). In the presence of codeine (COD), epinephrine (EP), acetaminophen (ACT), ascorbic acid (AA) or uric acid (UA) (38.45 μM), using Zn(II)-MOF/EDPGE, the anodic peak current measured for a 7.70 μM solution of SUM changes within ± 10%. Therefore, these compounds do not interfere with the measurement, and Zn(II)-MOF/EDPGE shows a reasonable selectivity for SUM.

### Study of the stability, reproducibility, and repeatability of the developed sensor

The stability of the sensor was studied by monitoring the current changes every two days. After a two-week storage period at room temperature, the sensor retained 94.25% of its original value, indicating an acceptable stability. To confirm the sensor's reproducibility, five modified electrodes (Zn(II)-MOF/EDPGE) were prepared for determination of SUM in a 7.70 μM solution using the same procedure. The resulting relative standard deviation (RSD) of 2.19% revealed that the proposed sensor exhibits good reproducibility. In addition, to examine the repeatability of the results measured with this sensor, four Zn(II)-MOF/EDPGE electrodes were used to detect the analyte under the same conditions. All four independent sensors exhibited similar responses, and the relative standard deviation (RSD) was 2.6%.

### Application of Zn(II)-MOF/EDPGE sensor for analysis of real samples

Measurement of SUM in human blood serum was conducted to evaluate the performance of the fabricated sensor in the analysis of real samples. Serum solutions with different concentrations of SUM were prepared according to the described method. The DPVs of the sensor were recorded for the prepared human blood serum samples, and the SUM concentrations were calculated using the corresponding calibration equation (Figs. S7A and B in the supplementary information). All measurements were carried out in triplicates, and the average current values were used in the calibration equation to calculate the standard deviations. By comparing the calculated concentrations with the actual concentrations, the recovery rate of the method for the solutions fell within the range of 96.6–111% (Table 3). According to the results, the fabricated Zn(II)-MOF/EDPGE sensor could successfully measure SUM in serum samples.

**Table 3:** Results obtained for the analysis of sumatriptan in human blood serum samples.

Sample	Spiked [μM]	Detected [μM]	Recovery (%)	RSD (%)
1	0	Not detected	–	–
2	0.99	0.98	99	2.3
3	1.99	2.21	111	3.6
4	2.99	2.89	96.6	2.4
5	4.98	5.011	100.6	1.5
6	5.96	6.12	102.6	2.4

## Conclusions

In this research study, (Zn(II)-MOF) was hydrothermally synthesized and characterized to evaluate its electrocatalytic performance. The compound was applied as a modifier of EDPGE to fabricate a highly sensitive electro-oxidation sumatriptan sensor. The bare PGE was electrochemically delaminated to enhance the surface porosity and introduce functional groups. Subsequently, a certain amount of the synthesized Zn(II)-MOF was cast onto the prepared EDPGE surface. The Zn(II)-MOF-modified EDPGE was used to detect and measure sumatriptan in standard buffer solution and human blood serum samples. The electrode surface was studied by SEM technique. Also, the electrochemical properties of the fabricated electrode were thoroughly investigated. The electrode exhibits a linear response to the 0.99–9.52 μM solutions of SUM in the standard phosphate buffer (pH = 7) and the detection limit was found to be 0.29 μM. Zn(II)-MOF catalyzes the electro-oxidation of SUM on the EDPGE surface. The synergistic effects of dual electrochemical and electrocatalytic modification of PGE enabled a rapid and sensitive determination of SUM. The fabricated Zn(II)-MOF/EDPGE electrochemical sensor represents advanced features including a high active surface area, excellent analytical characteristics, reasonable selectivity, high sensitivity, low cost, and satisfactory recovery values. These features depict the utility of Zn(II)-MOF/EDPGE as a simple, cost-effective, and rapid sensor for determination of SUM in real samples. We hope that the proposed strategy based on the fabricated electrode may aid in future efforts for routine measurements of the SUM in the real-world. Also this study can pave the way for new strategies in the fabrication of cost-effective sensor arrays to detect and measure other drugs.

### Supplementary Information

Supplementary Information 1.

Supplementary Information 2.

Supplementary Information 3.

## Data Availability

The data that support the findings of this study are available from the corresponding author upon request.

## Abbreviations

SUM: Sumatriptan
PGE: Pencil graphite electrode
GCE: Glassy carbon electrode
MWCNTs: Multi wall carbon nanotubes
MOFs: Metal-organic frameworks
SPCE: Screen-printed carbon electrodes
EDPGE: Electrochemically delaminated PGE
NH_2_BDC: 2-Amino-1,4-benzene dicarboxylic acid
KCl: Potassium chloride
DMF: Dimethylformamide
K_3_[Fe(CN)_6_]: Potassium hexacyanoferrate(III)
EIS: Electrochemical impedance spectroscopy
MSDS: Material safety data sheet
SEM: Scanning electron microscopy
CV: Cyclic voltammetry
DPV: Differential pulse voltammetry
COD: Codeine
EP: Epinephrine
ACT: Acetaminophen
AA: Ascorbic acid
UA: Uric acid
RSD: Relative standard deviation
