# Supplementary material for: Electro-oxidation sensing of sumatriptan in aqueous solutions and human blood serum by Zn(II)-MOF modified electrochemical delaminated pencil graphite electrode
Source: Sci Rep. 2023 Oct 5;13:16803. doi: 10.1038/s41598-023-44034-5 (PMC10556131; doi:10.1038/s41598-023-44034-5)
Supplement: Supplementary file 1 — Supplementary Information 1. [file 41598_2023_44034_MOESM1_ESM.htm]

Materials Studio Report


|  |
| --- |
| | Reflex |

# Reflex Summary Report for Rietveld Refinement of Zn-MOF

|  |  |  |  |
| --- | --- | --- | --- |
| Final Rwp: | 17.99% | Final Rp: | 13.95% |
| Final Rwp (without background): | 27.21% | Final CMACS: | 0.42% |

### Setup

|  |  |  |  |
| --- | --- | --- | --- |
| 2q Range (degrees): | 8.00-45.00 | Step Size (degrees): | 0.040 |
| Experiment: | exp-xrd.xcd |  |  |
| Excluded Regions: | - |  |  |

---

### Radiation

|  |  |  |  |
| --- | --- | --- | --- |
| Type: | X-ray | Source: | Copper |
| l1 (Å): | 1.540562 | l2 (Å): | 1.544390 |
| I2/I1: | 0.500 | Monochromator: | None |
| Anom. Dispersion: | No | Polarization: | 0.500 |

---

### Lattice Parameters

|  |  |  |  |
| --- | --- | --- | --- |
| Lattice Type: | Monoclinic | Space Group: | P 1 21/C 1 |

| Parameter | Value | Refined? |
| --- | --- | --- |
| a | 20.21525 ± *0.00685* | Yes |
| b | 31.33971 ± *0.00598* | Yes |
| c | 19.62813 ± *0.00784* | Yes |
| a | 90.00000 | No |
| b | 89.14761 ± *0.06117* | Yes |
| g | 90.00000 | No |

---

### Structure Parameters

|  |  |  |  |
| --- | --- | --- | --- |
| Refined Motion Groups: | 0 | Refined Torsions: | 0 |
| Refined Angles: | 0 | Refined Distances: | 0 |
| Number of Refined DOF: | 0 |  |  |

#### Fractional Coordinates

| No. | Name | u | v | w | Refined? |
| --- | --- | --- | --- | --- | --- |
| 1 | Zn1 | -0.27224 | 1.17882 | 0.44768 | No |
| 2 | Zn2 | -0.31891 | 1.07406 | 0.54813 | No |
| 3 | Zn3 | -0.33910 | 0.92652 | 0.04890 | No |
| 4 | Zn4 | -0.38718 | 0.82354 | -0.05529 | No |
| 5 | O1 | -0.26580 | 1.19830 | 0.54450 | No |
| 6 | O2 | -0.31100 | 1.13630 | 0.58330 | No |
| 7 | O3 | -0.25930 | 1.28630 | 0.86480 | No |
| 8 | O4 | -0.28030 | 1.22140 | 0.91180 | No |
| 9 | O5 | -0.27580 | 1.12090 | 0.39850 | No |
| 10 | O6 | -0.31870 | 1.06070 | 0.44850 | No |
| 11 | O7 | -0.33320 | 0.95120 | 0.14250 | No |
| 12 | O8 | -0.31890 | 1.01300 | 0.08570 | No |
| 13 | O9 | -0.33310 | 0.94310 | -0.04950 | No |
| 14 | O10 | -0.37970 | 0.88350 | -0.09790 | No |
| 15 | O11 | -0.31870 | 1.04750 | -0.35880 | No |
| 16 | O12 | -0.34160 | 0.98540 | -0.41410 | No |
| 17 | O13 | -0.39120 | 0.79330 | -0.14440 | No |
| 18 | O14 | -0.38780 | 0.72800 | -0.09350 | No |
| 19 | O15 | -0.34190 | 0.63560 | -0.41600 | No |
| 20 | O16 | -0.38580 | 0.69720 | -0.45940 | No |
| 21 | O17 | 0.07560 | 1.18670 | 0.55120 | No |
| 22 | O18 | 0.38130 | 1.14790 | 0.37050 | No |
| 23 | O19 | 0.02600 | 1.05200 | 0.45230 | No |
| 24 | O20 | 0.33530 | 1.09830 | 0.63320 | No |
| 25 | O21 | 0.00720 | 0.94810 | -0.04310 | No |
| 26 | O22 | 0.31660 | 0.90310 | 0.13600 | No |
| 27 | O23 | -0.03870 | 0.79410 | 0.03430 | No |
| 28 | O24 | 0.26490 | 0.84870 | -0.14420 | No |
| 29 | N1 | -0.21080 | 1.06660 | 0.54750 | No |
| 30 | N2 | -0.00230 | 1.06690 | 0.55750 | No |
| 31 | H2 | 0.01320 | 1.07370 | 0.59690 | No |
| 32 | N3 | 0.36660 | 1.06910 | 0.53170 | No |
| 33 | N4 | 0.57570 | 1.07700 | 0.54240 | No |
| 34 | N5 | -0.16400 | 1.17000 | 0.45520 | No |
| 35 | N6 | 0.04420 | 1.17060 | 0.44370 | No |
| 36 | H6 | 0.05880 | 1.16620 | 0.40350 | No |
| 37 | N7 | 0.41380 | 1.18320 | 0.46740 | No |
| 38 | H7 | 0.39970 | 1.19510 | 0.50400 | No |
| 39 | N8 | 0.62140 | 1.18260 | 0.45060 | No |
| 40 | N9 | -0.23150 | 0.92180 | 0.04690 | No |
| 41 | N10 | -0.02430 | 0.91730 | 0.05470 | No |
| 42 | H10 | -0.00860 | 0.90550 | 0.09110 | No |
| 43 | N11 | 0.34700 | 0.93370 | 0.03420 | No |
| 44 | H11 | 0.33190 | 0.94430 | -0.00330 | No |
| 45 | N12 | 0.55330 | 0.93170 | 0.04590 | No |
| 46 | N13 | -0.27860 | 0.81790 | -0.05500 | No |
| 47 | N14 | -0.07110 | 0.81630 | -0.06900 | No |
| 48 | H14 | -0.05640 | 0.82460 | -0.10770 | No |
| 49 | N15 | 0.29860 | 0.82340 | -0.04230 | No |
| 50 | H15 | 0.28480 | 0.81350 | -0.00410 | No |
| 51 | N16 | 0.50630 | 0.82930 | -0.04900 | No |
| 52 | C1 | -0.27670 | 1.18990 | 0.66150 | No |
| 53 | C2 | -0.25750 | 1.23360 | 0.67170 | No |
| 54 | H2A | -0.24640 | 1.25090 | 0.63460 | No |
| 55 | C3 | -0.25460 | 1.25170 | 0.73600 | No |
| 56 | H3 | -0.24250 | 1.28100 | 0.74190 | No |
| 57 | C4 | -0.26990 | 1.22590 | 0.79170 | No |
| 58 | C5 | -0.28760 | 1.18190 | 0.78200 | No |
| 59 | H5 | -0.29650 | 1.16420 | 0.81940 | No |
| 60 | C6 | -0.29210 | 1.16400 | 0.71740 | No |
| 61 | H6A | -0.30530 | 1.13480 | 0.71150 | No |
| 62 | C7 | -0.28490 | 1.17320 | 0.59100 | No |
| 63 | C8 | -0.26910 | 1.24530 | 0.86290 | No |
| 64 | C9 | -0.30250 | 1.05960 | 0.33070 | No |
| 65 | C10 | -0.29500 | 1.08300 | 0.27010 | No |
| 66 | H10A | -0.28650 | 1.11310 | 0.27060 | No |
| 67 | C11 | -0.30050 | 1.06020 | 0.20910 | No |
| 68 | H11A | -0.29550 | 1.07560 | 0.16870 | No |
| 69 | C12 | -0.31300 | 1.01630 | 0.20660 | No |
| 70 | C13 | -0.31880 | 0.99250 | 0.26590 | No |
| 71 | H13 | -0.32490 | 0.96210 | 0.26440 | No |
| 72 | C14 | -0.31560 | 1.01430 | 0.32790 | No |
| 73 | H14A | -0.32220 | 0.99860 | 0.36770 | No |
| 74 | C15 | -0.29930 | 1.08200 | 0.39780 | No |
| 75 | C16 | -0.32160 | 0.99320 | 0.13900 | No |
| 76 | C17 | -0.34360 | 0.94160 | -0.16810 | No |
| 77 | C18 | -0.35680 | 0.91810 | -0.22810 | No |
| 78 | H18 | -0.36910 | 0.88850 | -0.22630 | No |
| 79 | C19 | -0.35120 | 0.93940 | -0.28990 | No |
| 80 | H19 | -0.36050 | 0.92400 | -0.32970 | No |
| 81 | C20 | -0.33190 | 0.98340 | -0.29310 | No |
| 82 | C21 | -0.31810 | 1.00630 | -0.23460 | No |
| 83 | H21 | -0.30540 | 1.03570 | -0.23690 | No |
| 84 | C22 | -0.32290 | 0.98570 | -0.17260 | No |
| 85 | H22 | -0.31220 | 1.00120 | -0.13330 | No |
| 86 | C23 | -0.35150 | 0.92110 | -0.09990 | No |
| 87 | C24 | -0.33050 | 1.00650 | -0.36180 | No |
| 88 | C25 | -0.37750 | 0.73150 | -0.21300 | No |
| 89 | C26 | -0.38320 | 0.75770 | -0.27150 | No |
| 90 | H26 | -0.38890 | 0.78810 | -0.26780 | No |
| 91 | C27 | -0.38020 | 0.73810 | -0.33480 | No |
| 92 | H27 | -0.38650 | 0.75520 | -0.37350 | No |
| 93 | C28 | -0.36800 | 0.69350 | -0.34070 | No |
| 94 | C29 | -0.35940 | 0.66730 | -0.28340 | No |
| 95 | H29 | -0.34970 | 0.63740 | -0.28750 | No |
| 96 | C30 | -0.36570 | 0.68640 | -0.22000 | No |
| 97 | H30 | -0.36200 | 0.66880 | -0.18150 | No |
| 98 | C31 | -0.38570 | 0.75180 | -0.14450 | No |
| 99 | C32 | -0.36550 | 0.67330 | -0.41040 | No |
| 100 | C33 | -0.17420 | 1.05460 | 0.49550 | No |
| 101 | H33 | -0.19630 | 1.04620 | 0.45590 | No |
| 102 | C34 | -0.10600 | 1.05350 | 0.49530 | No |
| 103 | H34 | -0.08330 | 1.04500 | 0.45660 | No |
| 104 | C35 | -0.07220 | 1.06520 | 0.55290 | No |
| 105 | C36 | -0.11000 | 1.07860 | 0.60880 | No |
| 106 | H36 | -0.08890 | 1.08710 | 0.64880 | No |
| 107 | C37 | -0.17720 | 1.07920 | 0.60420 | No |
| 108 | H37 | -0.20110 | 1.08860 | 0.64150 | No |
| 109 | C38 | 0.04230 | 1.06000 | 0.51180 | No |
| 110 | C39 | 0.11360 | 1.06350 | 0.53100 | No |
| 111 | C40 | 0.13510 | 1.07430 | 0.59480 | No |
| 112 | H40 | 0.10410 | 1.07880 | 0.62860 | No |
| 113 | C41 | 0.20240 | 1.07880 | 0.61060 | No |
| 114 | H41 | 0.21650 | 1.08520 | 0.65450 | No |
| 115 | C42 | 0.24980 | 1.07330 | 0.55660 | No |
| 116 | C43 | 0.22720 | 1.06320 | 0.49500 | No |
| 117 | H43 | 0.25720 | 1.05910 | 0.46020 | No |
| 118 | C44 | 0.15980 | 1.05860 | 0.48130 | No |
| 119 | H44 | 0.14570 | 1.05210 | 0.43740 | No |
| 120 | C45 | 0.31990 | 1.08190 | 0.58000 | No |
| 121 | C46 | 0.53870 | 1.09400 | 0.59220 | No |
| 122 | H46 | 0.56060 | 1.10680 | 0.62890 | No |
| 123 | C47 | 0.47100 | 1.09380 | 0.59230 | No |
| 124 | H47 | 0.44820 | 1.10740 | 0.62730 | No |
| 125 | C48 | 0.43740 | 1.07370 | 0.54140 | No |
| 126 | C49 | 0.47370 | 1.05540 | 0.48770 | No |
| 127 | H49 | 0.45230 | 1.04250 | 0.45080 | No |
| 128 | C50 | 0.54080 | 1.05720 | 0.49130 | No |
| 129 | H50 | 0.56450 | 1.04400 | 0.45660 | No |
| 130 | C51 | -0.13060 | 1.15720 | 0.40030 | No |
| 131 | H51 | -0.15500 | 1.14840 | 0.36240 | No |
| 132 | C52 | -0.06400 | 1.15630 | 0.39620 | No |
| 133 | H52 | -0.04410 | 1.14730 | 0.35620 | No |
| 134 | C53 | -0.02440 | 1.16900 | 0.45180 | No |
| 135 | C54 | -0.05820 | 1.18060 | 0.50890 | No |
| 136 | H54 | -0.03520 | 1.18860 | 0.54810 | No |
| 137 | C55 | -0.12700 | 1.18040 | 0.50790 | No |
| 138 | H55 | -0.14850 | 1.18810 | 0.54760 | No |
| 139 | C56 | 0.09040 | 1.17840 | 0.49360 | No |
| 140 | C57 | 0.16030 | 1.17650 | 0.47270 | No |
| 141 | C58 | 0.20890 | 1.18310 | 0.52330 | No |
| 142 | H58 | 0.19590 | 1.18920 | 0.56740 | No |
| 143 | C59 | 0.27600 | 1.18080 | 0.50850 | No |
| 144 | H59 | 0.30730 | 1.18520 | 0.54280 | No |
| 145 | C60 | 0.29640 | 1.17220 | 0.44470 | No |
| 146 | C61 | 0.25120 | 1.16440 | 0.39430 | No |
| 147 | H61 | 0.26500 | 1.15680 | 0.35110 | No |
| 148 | C62 | 0.18310 | 1.16800 | 0.40870 | No |
| 149 | H62 | 0.15240 | 1.16440 | 0.37360 | No |
| 150 | C63 | 0.36820 | 1.16580 | 0.42280 | No |
| 151 | C64 | 0.58410 | 1.16430 | 0.40140 | No |
| 152 | H64 | 0.60540 | 1.15250 | 0.36400 | No |
| 153 | C65 | 0.51450 | 1.16210 | 0.40340 | No |
| 154 | H65 | 0.49060 | 1.14720 | 0.36990 | No |
| 155 | C66 | 0.48250 | 1.18250 | 0.45600 | No |
| 156 | C67 | 0.52160 | 1.20490 | 0.50500 | No |
| 157 | H67 | 0.50180 | 1.21960 | 0.54070 | No |
| 158 | C68 | 0.58960 | 1.20460 | 0.49910 | No |
| 159 | H68 | 0.61460 | 1.22050 | 0.53050 | No |
| 160 | C69 | -0.19400 | 0.91090 | 0.10410 | No |
| 161 | H69 | -0.21570 | 0.90350 | 0.14380 | No |
| 162 | C70 | -0.13030 | 0.91070 | 0.10390 | No |
| 163 | H70 | -0.10910 | 0.90490 | 0.14500 | No |
| 164 | C69' | -0.19900 | 0.89770 | 0.09290 | No |
| 165 | H69' | -0.22390 | 0.88160 | 0.12360 | No |
| 166 | C70' | -0.13060 | 0.89500 | 0.09820 | No |
| 167 | H70' | -0.11080 | 0.87840 | 0.13240 | No |
| 168 | C71 | -0.09170 | 0.91780 | 0.05120 | No |
| 169 | C72 | -0.12410 | 0.92610 | -0.00560 | No |
| 170 | H72 | -0.10090 | 0.93000 | -0.04560 | No |
| 171 | C73 | -0.19290 | 0.92900 | -0.00610 | No |
| 172 | H73 | -0.21360 | 0.93650 | -0.04680 | No |
| 173 | C72' | -0.12630 | 0.94920 | 0.00320 | No |
| 174 | H72' | -0.10350 | 0.96790 | -0.02560 | No |
| 175 | C73' | -0.19420 | 0.94780 | 0.00530 | No |
| 176 | H73' | -0.21710 | 0.96620 | -0.02450 | No |
| 177 | C74 | 0.02240 | 0.93180 | 0.01250 | No |
| 178 | C75 | 0.09250 | 0.92760 | 0.02960 | No |
| 179 | C76 | 0.14150 | 0.93100 | -0.01580 | No |
| 180 | H76 | 0.12880 | 0.93470 | -0.06090 | No |
| 181 | C77 | 0.20740 | 0.92940 | -0.00220 | No |
| 182 | H77 | 0.23720 | 0.93290 | -0.03730 | No |
| 183 | C76' | 0.14120 | 0.95740 | -0.00440 | No |
| 184 | H76' | 0.12640 | 0.97870 | -0.03490 | No |
| 185 | C77' | 0.20500 | 0.95330 | 0.00920 | No |
| 186 | H77' | 0.23500 | 0.97100 | -0.01390 | No |
| 187 | C78 | 0.22920 | 0.92310 | 0.05760 | No |
| 188 | C79 | 0.18390 | 0.91640 | 0.11490 | No |
| 189 | H79 | 0.19930 | 0.91180 | 0.15900 | No |
| 190 | C80 | 0.11630 | 0.91730 | 0.09850 | No |
| 191 | H80 | 0.08560 | 0.91130 | 0.13200 | No |
| 192 | C79' | 0.18430 | 0.89620 | 0.08920 | No |
| 193 | H79' | 0.19900 | 0.87600 | 0.12140 | No |
| 194 | C80' | 0.11670 | 0.89900 | 0.07370 | No |
| 195 | H80' | 0.08770 | 0.87990 | 0.09520 | No |
| 196 | C81 | 0.30120 | 0.91840 | 0.08180 | No |
| 197 | C82 | 0.51970 | 0.94510 | -0.00860 | No |
| 198 | H82 | 0.54340 | 0.95400 | -0.04630 | No |
| 199 | C83 | 0.45200 | 0.94630 | -0.01210 | No |
| 200 | H83 | 0.43070 | 0.95620 | -0.05130 | No |
| 201 | C84 | 0.41480 | 0.93260 | 0.04410 | No |
| 202 | C85 | 0.45020 | 0.91870 | 0.10140 | No |
| 203 | H85 | 0.42790 | 0.90940 | 0.13970 | No |
| 204 | C86 | 0.51770 | 0.91890 | 0.10070 | No |
| 205 | H86 | 0.54060 | 0.90990 | 0.13940 | No |
| 206 | C87 | -0.24060 | 0.80040 | -0.00770 | No |
| 207 | H87 | -0.26150 | 0.78820 | 0.02970 | No |
| 208 | C88 | -0.17260 | 0.79860 | -0.00880 | No |
| 209 | H88 | -0.14910 | 0.78620 | 0.02730 | No |
| 210 | C89 | -0.13970 | 0.81500 | -0.06320 | No |
| 211 | C90 | -0.17860 | 0.82400 | -0.12370 | No |
| 212 | C91 | -0.24740 | 0.83890 | -0.10730 | No |
| 213 | C90' | -0.17960 | 0.84120 | -0.10920 | No |
| 214 | C91' | -0.24660 | 0.82340 | -0.11390 | No |
| 215 | C92 | -0.02500 | 0.80620 | -0.02190 | No |
| 216 | C93 | 0.04640 | 0.81100 | -0.04390 | No |
| 217 | C94 | 0.09300 | 0.80430 | 0.00310 | No |
| 218 | H94 | 0.08120 | 0.79530 | 0.04630 | No |
| 219 | C95 | 0.16080 | 0.81090 | -0.01280 | No |
| 220 | H95 | 0.19240 | 0.80460 | 0.02060 | No |
| 221 | C94' | 0.09470 | 0.79000 | -0.00580 | No |
| 222 | H94' | 0.08160 | 0.77140 | 0.02910 | No |
| 223 | C95' | 0.16070 | 0.79510 | -0.01720 | No |
| 224 | H95' | 0.19200 | 0.77970 | 0.00840 | No |
| 225 | C96 | 0.18110 | 0.82480 | -0.06930 | No |
| 226 | C97 | 0.13220 | 0.83170 | -0.12290 | No |
| 227 | H97 | 0.14560 | 0.83930 | -0.16620 | No |
| 228 | C98 | 0.06590 | 0.82680 | -0.10920 | No |
| 229 | H98 | 0.03410 | 0.83360 | -0.14190 | No |
| 230 | C97' | 0.13160 | 0.85050 | -0.10450 | No |
| 231 | H97' | 0.14410 | 0.87120 | -0.13680 | No |
| 232 | C98' | 0.06550 | 0.84440 | -0.08990 | No |
| 233 | H98' | 0.03350 | 0.86220 | -0.11030 | No |
| 234 | C99 | 0.25210 | 0.83350 | -0.09000 | No |
| 235 | C100 | 0.47010 | 0.83950 | -0.10430 | No |
| 236 | H100 | 0.49240 | 0.84730 | -0.14350 | No |
| 237 | C101 | 0.39980 | 0.83910 | -0.10620 | No |
| 238 | H101 | 0.37670 | 0.84660 | -0.14560 | No |
| 239 | C102 | 0.36760 | 0.82770 | -0.04990 | No |
| 240 | C103 | 0.40510 | 0.81780 | 0.00970 | No |
| 241 | H103 | 0.38390 | 0.81060 | 0.04980 | No |
| 242 | C104 | 0.47280 | 0.81890 | 0.00730 | No |
| 243 | H104 | 0.49690 | 0.81210 | 0.04640 | No |
| 244 | O1S | 0.53360 | 0.39110 | 0.75710 | No |
| 245 | N1S | 0.51660 | 0.46620 | 0.75640 | No |
| 246 | C1S | 0.52560 | 0.42850 | 0.78730 | No |
| 247 | H1S | 0.52650 | 0.42860 | 0.83440 | No |
| 248 | C2S | 0.51120 | 0.47160 | 0.68730 | No |
| 249 | H2S1 | 0.52050 | 0.44410 | 0.66520 | No |
| 250 | H2S2 | 0.54220 | 0.49360 | 0.67280 | No |
| 251 | H2S3 | 0.46700 | 0.48090 | 0.67570 | No |
| 252 | C3S | 0.50760 | 0.50530 | 0.79660 | No |
| 253 | H3S1 | 0.46230 | 0.51480 | 0.79310 | No |
| 254 | H3S2 | 0.53620 | 0.52820 | 0.78050 | No |
| 255 | H3S3 | 0.51840 | 0.49890 | 0.84310 | No |
| 256 | O2S | 0.47250 | 0.63720 | 0.75180 | No |
| 257 | N2S | 0.45350 | 0.71190 | 0.74650 | No |
| 258 | C4S | 0.46380 | 0.66940 | 0.72070 | No |
| 259 | H4S | 0.46360 | 0.66650 | 0.67380 | No |
| 260 | C5S | 0.44400 | 0.74510 | 0.70030 | No |
| 261 | H5S1 | 0.46480 | 0.77160 | 0.71670 | No |
| 262 | H5S2 | 0.39740 | 0.75010 | 0.69390 | No |
| 263 | H5S3 | 0.46330 | 0.73680 | 0.65790 | No |
| 264 | C6S | 0.45200 | 0.71920 | 0.81620 | No |
| 265 | H6S1 | 0.45620 | 0.75020 | 0.82500 | No |
| 266 | H6S2 | 0.48800 | 0.70370 | 0.83770 | No |
| 267 | H6S3 | 0.41080 | 0.70870 | 0.83370 | No |
| 268 | O3S | 0.03530 | 0.90330 | 0.75570 | No |
| 269 | N3S | 0.09740 | 0.96660 | 0.76070 | No |
| 270 | C7S | 0.06740 | 0.94240 | 0.78600 | No |
| 271 | H7S | 0.06110 | 0.94660 | 0.83210 | No |
| 272 | C8S | 0.12020 | 0.96450 | 0.69770 | No |
| 273 | H8S1 | 0.16730 | 0.96860 | 0.69840 | No |
| 274 | H8S2 | 0.09980 | 0.98720 | 0.67070 | No |
| 275 | H8S3 | 0.10980 | 0.93610 | 0.67860 | No |
| 276 | C9S | 0.11540 | 1.00370 | 0.80610 | No |
| 277 | H9S1 | 0.09820 | 0.99820 | 0.85040 | No |
| 278 | H9S2 | 0.09700 | 1.03060 | 0.78850 | No |
| 279 | H9S3 | 0.16280 | 1.00620 | 0.80890 | No |
| 280 | O4S | 0.00090 | 1.13850 | 0.81440 | No |
| 281 | N4S | -0.06090 | 1.20450 | 0.78440 | No |
| 282 | C10S | -0.01940 | 1.18020 | 0.82760 | No |
| 283 | H10S | -0.00490 | 1.19340 | 0.86760 | No |
| 284 | C11S | -0.06220 | 1.25180 | 0.79630 | No |
| 285 | H11B | -0.10160 | 1.25940 | 0.82040 | No |
| 286 | H11C | -0.06180 | 1.26710 | 0.75370 | No |
| 287 | H11D | -0.02390 | 1.26020 | 0.82270 | No |
| 288 | C12S | -0.09420 | 1.18820 | 0.72440 | No |
| 289 | H12A | -0.06330 | 1.18660 | 0.68790 | No |
| 290 | H12B | -0.12970 | 1.20790 | 0.71210 | No |
| 291 | H12C | -0.11190 | 1.15940 | 0.73310 | No |
| 292 | O5S | -0.06380 | 0.76980 | -0.21610 | No |
| 293 | N5S | -0.14680 | 0.71180 | -0.24030 | No |
| 294 | C13S | -0.09300 | 0.72950 | -0.20370 | No |
| 295 | H13S | -0.07570 | 0.71270 | -0.16830 | No |
| 296 | C14S | -0.14540 | 0.66850 | -0.22250 | No |
| 297 | H14B | -0.10770 | 0.65460 | -0.24240 | No |
| 298 | H14C | -0.18520 | 0.65430 | -0.23830 | No |
| 299 | H14D | -0.14240 | 0.66610 | -0.17410 | No |
| 300 | C15S | -0.17410 | 0.71800 | -0.30620 | No |
| 301 | H15A | -0.20190 | 0.69330 | -0.31780 | No |
| 302 | H15B | -0.13900 | 0.72040 | -0.33840 | No |
| 303 | H15C | -0.20000 | 0.74460 | -0.30710 | No |
| 304 | O6S | -0.06690 | 0.84160 | -0.29430 | No |
| 305 | O7S | 0.02160 | 1.08400 | 0.70800 | No |
| 306 | O8S | -0.11790 | 0.96140 | 0.75950 | No |
| 307 | H | 0.84717 | 0.66923 | 0.32630 | No |
| 308 | H | 0.72211 | 0.64532 | 0.35018 | No |
| 309 | H | 0.84526 | 0.63609 | 0.35271 | No |
| 310 | H | 0.72400 | 0.67173 | 0.33701 | No |
| 311 | H | 0.65018 | 0.94547 | 0.51716 | No |

#### Occupancies

| No. | Name | Occupancy | Refined? |
| --- | --- | --- | --- |
| 1 | Zn1 | 1.00000 | No |
| 2 | Zn2 | 1.00000 | No |
| 3 | Zn3 | 1.00000 | No |
| 4 | Zn4 | 1.00000 | No |
| 5 | O1 | 1.00000 | No |
| 6 | O2 | 1.00000 | No |
| 7 | O3 | 1.00000 | No |
| 8 | O4 | 1.00000 | No |
| 9 | O5 | 1.00000 | No |
| 10 | O6 | 1.00000 | No |
| 11 | O7 | 1.00000 | No |
| 12 | O8 | 1.00000 | No |
| 13 | O9 | 1.00000 | No |
| 14 | O10 | 1.00000 | No |
| 15 | O11 | 1.00000 | No |
| 16 | O12 | 1.00000 | No |
| 17 | O13 | 1.00000 | No |
| 18 | O14 | 1.00000 | No |
| 19 | O15 | 1.00000 | No |
| 20 | O16 | 1.00000 | No |
| 21 | O17 | 1.00000 | No |
| 22 | O18 | 1.00000 | No |
| 23 | O19 | 1.00000 | No |
| 24 | O20 | 1.00000 | No |
| 25 | O21 | 1.00000 | No |
| 26 | O22 | 1.00000 | No |
| 27 | O23 | 1.00000 | No |
| 28 | O24 | 1.00000 | No |
| 29 | N1 | 1.00000 | No |
| 30 | N2 | 1.00000 | No |
| 31 | H2 | 1.00000 | No |
| 32 | N3 | 1.00000 | No |
| 33 | N4 | 1.00000 | No |
| 34 | N5 | 1.00000 | No |
| 35 | N6 | 1.00000 | No |
| 36 | H6 | 1.00000 | No |
| 37 | N7 | 1.00000 | No |
| 38 | H7 | 1.00000 | No |
| 39 | N8 | 1.00000 | No |
| 40 | N9 | 1.00000 | No |
| 41 | N10 | 1.00000 | No |
| 42 | H10 | 1.00000 | No |
| 43 | N11 | 1.00000 | No |
| 44 | H11 | 1.00000 | No |
| 45 | N12 | 1.00000 | No |
| 46 | N13 | 1.00000 | No |
| 47 | N14 | 1.00000 | No |
| 48 | H14 | 1.00000 | No |
| 49 | N15 | 1.00000 | No |
| 50 | H15 | 1.00000 | No |
| 51 | N16 | 1.00000 | No |
| 52 | C1 | 1.00000 | No |
| 53 | C2 | 1.00000 | No |
| 54 | H2A | 1.00000 | No |
| 55 | C3 | 1.00000 | No |
| 56 | H3 | 1.00000 | No |
| 57 | C4 | 1.00000 | No |
| 58 | C5 | 1.00000 | No |
| 59 | H5 | 1.00000 | No |
| 60 | C6 | 1.00000 | No |
| 61 | H6A | 1.00000 | No |
| 62 | C7 | 1.00000 | No |
| 63 | C8 | 1.00000 | No |
| 64 | C9 | 1.00000 | No |
| 65 | C10 | 1.00000 | No |
| 66 | H10A | 1.00000 | No |
| 67 | C11 | 1.00000 | No |
| 68 | H11A | 1.00000 | No |
| 69 | C12 | 1.00000 | No |
| 70 | C13 | 1.00000 | No |
| 71 | H13 | 1.00000 | No |
| 72 | C14 | 1.00000 | No |
| 73 | H14A | 1.00000 | No |
| 74 | C15 | 1.00000 | No |
| 75 | C16 | 1.00000 | No |
| 76 | C17 | 1.00000 | No |
| 77 | C18 | 1.00000 | No |
| 78 | H18 | 1.00000 | No |
| 79 | C19 | 1.00000 | No |
| 80 | H19 | 1.00000 | No |
| 81 | C20 | 1.00000 | No |
| 82 | C21 | 1.00000 | No |
| 83 | H21 | 1.00000 | No |
| 84 | C22 | 1.00000 | No |
| 85 | H22 | 1.00000 | No |
| 86 | C23 | 1.00000 | No |
| 87 | C24 | 1.00000 | No |
| 88 | C25 | 1.00000 | No |
| 89 | C26 | 1.00000 | No |
| 90 | H26 | 1.00000 | No |
| 91 | C27 | 1.00000 | No |
| 92 | H27 | 1.00000 | No |
| 93 | C28 | 1.00000 | No |
| 94 | C29 | 1.00000 | No |
| 95 | H29 | 1.00000 | No |
| 96 | C30 | 1.00000 | No |
| 97 | H30 | 1.00000 | No |
| 98 | C31 | 1.00000 | No |
| 99 | C32 | 1.00000 | No |
| 100 | C33 | 1.00000 | No |
| 101 | H33 | 1.00000 | No |
| 102 | C34 | 1.00000 | No |
| 103 | H34 | 1.00000 | No |
| 104 | C35 | 1.00000 | No |
| 105 | C36 | 1.00000 | No |
| 106 | H36 | 1.00000 | No |
| 107 | C37 | 1.00000 | No |
| 108 | H37 | 1.00000 | No |
| 109 | C38 | 1.00000 | No |
| 110 | C39 | 1.00000 | No |
| 111 | C40 | 1.00000 | No |
| 112 | H40 | 1.00000 | No |
| 113 | C41 | 1.00000 | No |
| 114 | H41 | 1.00000 | No |
| 115 | C42 | 1.00000 | No |
| 116 | C43 | 1.00000 | No |
| 117 | H43 | 1.00000 | No |
| 118 | C44 | 1.00000 | No |
| 119 | H44 | 1.00000 | No |
| 120 | C45 | 1.00000 | No |
| 121 | C46 | 1.00000 | No |
| 122 | H46 | 1.00000 | No |
| 123 | C47 | 1.00000 | No |
| 124 | H47 | 1.00000 | No |
| 125 | C48 | 1.00000 | No |
| 126 | C49 | 1.00000 | No |
| 127 | H49 | 1.00000 | No |
| 128 | C50 | 1.00000 | No |
| 129 | H50 | 1.00000 | No |
| 130 | C51 | 1.00000 | No |
| 131 | H51 | 1.00000 | No |
| 132 | C52 | 1.00000 | No |
| 133 | H52 | 1.00000 | No |
| 134 | C53 | 1.00000 | No |
| 135 | C54 | 1.00000 | No |
| 136 | H54 | 1.00000 | No |
| 137 | C55 | 1.00000 | No |
| 138 | H55 | 1.00000 | No |
| 139 | C56 | 1.00000 | No |
| 140 | C57 | 1.00000 | No |
| 141 | C58 | 1.00000 | No |
| 142 | H58 | 1.00000 | No |
| 143 | C59 | 1.00000 | No |
| 144 | H59 | 1.00000 | No |
| 145 | C60 | 1.00000 | No |
| 146 | C61 | 1.00000 | No |
| 147 | H61 | 1.00000 | No |
| 148 | C62 | 1.00000 | No |
| 149 | H62 | 1.00000 | No |
| 150 | C63 | 1.00000 | No |
| 151 | C64 | 1.00000 | No |
| 152 | H64 | 1.00000 | No |
| 153 | C65 | 1.00000 | No |
| 154 | H65 | 1.00000 | No |
| 155 | C66 | 1.00000 | No |
| 156 | C67 | 1.00000 | No |
| 157 | H67 | 1.00000 | No |
| 158 | C68 | 1.00000 | No |
| 159 | H68 | 1.00000 | No |
| 160 | C69 | 0.50000 | No |
| 161 | H69 | 0.50000 | No |
| 162 | C70 | 0.50000 | No |
| 163 | H70 | 0.50000 | No |
| 164 | C69' | 0.50000 | No |
| 165 | H69' | 0.50000 | No |
| 166 | C70' | 0.50000 | No |
| 167 | H70' | 0.50000 | No |
| 168 | C71 | 1.00000 | No |
| 169 | C72 | 0.50000 | No |
| 170 | H72 | 0.50000 | No |
| 171 | C73 | 0.50000 | No |
| 172 | H73 | 0.50000 | No |
| 173 | C72' | 0.50000 | No |
| 174 | H72' | 0.50000 | No |
| 175 | C73' | 0.50000 | No |
| 176 | H73' | 0.50000 | No |
| 177 | C74 | 1.00000 | No |
| 178 | C75 | 1.00000 | No |
| 179 | C76 | 0.50000 | No |
| 180 | H76 | 0.50000 | No |
| 181 | C77 | 0.50000 | No |
| 182 | H77 | 0.50000 | No |
| 183 | C76' | 0.50000 | No |
| 184 | H76' | 0.50000 | No |
| 185 | C77' | 0.50000 | No |
| 186 | H77' | 0.50000 | No |
| 187 | C78 | 1.00000 | No |
| 188 | C79 | 0.50000 | No |
| 189 | H79 | 0.50000 | No |
| 190 | C80 | 0.50000 | No |
| 191 | H80 | 0.50000 | No |
| 192 | C79' | 0.50000 | No |
| 193 | H79' | 0.50000 | No |
| 194 | C80' | 0.50000 | No |
| 195 | H80' | 0.50000 | No |
| 196 | C81 | 1.00000 | No |
| 197 | C82 | 1.00000 | No |
| 198 | H82 | 1.00000 | No |
| 199 | C83 | 1.00000 | No |
| 200 | H83 | 1.00000 | No |
| 201 | C84 | 1.00000 | No |
| 202 | C85 | 1.00000 | No |
| 203 | H85 | 1.00000 | No |
| 204 | C86 | 1.00000 | No |
| 205 | H86 | 1.00000 | No |
| 206 | C87 | 1.00000 | No |
| 207 | H87 | 1.00000 | No |
| 208 | C88 | 1.00000 | No |
| 209 | H88 | 1.00000 | No |
| 210 | C89 | 1.00000 | No |
| 211 | C90 | 0.50000 | No |
| 212 | C91 | 0.50000 | No |
| 213 | C90' | 0.50000 | No |
| 214 | C91' | 0.50000 | No |
| 215 | C92 | 1.00000 | No |
| 216 | C93 | 1.00000 | No |
| 217 | C94 | 0.50000 | No |
| 218 | H94 | 0.50000 | No |
| 219 | C95 | 0.50000 | No |
| 220 | H95 | 0.50000 | No |
| 221 | C94' | 0.50000 | No |
| 222 | H94' | 0.50000 | No |
| 223 | C95' | 0.50000 | No |
| 224 | H95' | 0.50000 | No |
| 225 | C96 | 1.00000 | No |
| 226 | C97 | 0.50000 | No |
| 227 | H97 | 0.50000 | No |
| 228 | C98 | 0.50000 | No |
| 229 | H98 | 0.50000 | No |
| 230 | C97' | 0.50000 | No |
| 231 | H97' | 0.50000 | No |
| 232 | C98' | 0.50000 | No |
| 233 | H98' | 0.50000 | No |
| 234 | C99 | 1.00000 | No |
| 235 | C100 | 1.00000 | No |
| 236 | H100 | 1.00000 | No |
| 237 | C101 | 1.00000 | No |
| 238 | H101 | 1.00000 | No |
| 239 | C102 | 1.00000 | No |
| 240 | C103 | 1.00000 | No |
| 241 | H103 | 1.00000 | No |
| 242 | C104 | 1.00000 | No |
| 243 | H104 | 1.00000 | No |
| 244 | O1S | 1.00000 | No |
| 245 | N1S | 1.00000 | No |
| 246 | C1S | 1.00000 | No |
| 247 | H1S | 1.00000 | No |
| 248 | C2S | 1.00000 | No |
| 249 | H2S1 | 1.00000 | No |
| 250 | H2S2 | 1.00000 | No |
| 251 | H2S3 | 1.00000 | No |
| 252 | C3S | 1.00000 | No |
| 253 | H3S1 | 1.00000 | No |
| 254 | H3S2 | 1.00000 | No |
| 255 | H3S3 | 1.00000 | No |
| 256 | O2S | 1.00000 | No |
| 257 | N2S | 1.00000 | No |
| 258 | C4S | 1.00000 | No |
| 259 | H4S | 1.00000 | No |
| 260 | C5S | 1.00000 | No |
| 261 | H5S1 | 1.00000 | No |
| 262 | H5S2 | 1.00000 | No |
| 263 | H5S3 | 1.00000 | No |
| 264 | C6S | 1.00000 | No |
| 265 | H6S1 | 1.00000 | No |
| 266 | H6S2 | 1.00000 | No |
| 267 | H6S3 | 1.00000 | No |
| 268 | O3S | 1.00000 | No |
| 269 | N3S | 1.00000 | No |
| 270 | C7S | 1.00000 | No |
| 271 | H7S | 1.00000 | No |
| 272 | C8S | 1.00000 | No |
| 273 | H8S1 | 1.00000 | No |
| 274 | H8S2 | 1.00000 | No |
| 275 | H8S3 | 1.00000 | No |
| 276 | C9S | 1.00000 | No |
| 277 | H9S1 | 1.00000 | No |
| 278 | H9S2 | 1.00000 | No |
| 279 | H9S3 | 1.00000 | No |
| 280 | O4S | 1.00000 | No |
| 281 | N4S | 1.00000 | No |
| 282 | C10S | 1.00000 | No |
| 283 | H10S | 1.00000 | No |
| 284 | C11S | 1.00000 | No |
| 285 | H11B | 1.00000 | No |
| 286 | H11C | 1.00000 | No |
| 287 | H11D | 1.00000 | No |
| 288 | C12S | 1.00000 | No |
| 289 | H12A | 1.00000 | No |
| 290 | H12B | 1.00000 | No |
| 291 | H12C | 1.00000 | No |
| 292 | O5S | 1.00000 | No |
| 293 | N5S | 1.00000 | No |
| 294 | C13S | 1.00000 | No |
| 295 | H13S | 1.00000 | No |
| 296 | C14S | 1.00000 | No |
| 297 | H14B | 1.00000 | No |
| 298 | H14C | 1.00000 | No |
| 299 | H14D | 1.00000 | No |
| 300 | C15S | 1.00000 | No |
| 301 | H15A | 1.00000 | No |
| 302 | H15B | 1.00000 | No |
| 303 | H15C | 1.00000 | No |
| 304 | O6S | 1.00000 | No |
| 305 | O7S | 1.00000 | No |
| 306 | O8S | 1.00000 | No |
| 307 | H | 0.50000 | No |
| 308 | H | 0.50000 | No |
| 309 | H | 0.50000 | No |
| 310 | H | 0.50000 | No |
| 311 | H | 1.00000 | No |

#### Temperature Factors

| No. | Name | Ueq | U11 | U22 | U33 | U12 | U23 | U13 | Refined? |
| --- | --- | --- | --- | --- | --- | --- | --- | --- | --- |
| 1 | Zn1 | 0.02342 | 0.02528 | 0.02837 | 0.01660 | -0.00145 | -0.00093 | -0.00020 | No |
| 2 | Zn2 | 0.02197 | 0.02358 | 0.02666 | 0.01571 | -0.00135 | 0.00021 | -0.00189 | No |
| 3 | Zn3 | 0.02336 | 0.02749 | 0.02677 | 0.01581 | -0.00249 | 0.00113 | -0.00030 | No |
| 4 | Zn4 | 0.02330 | 0.02418 | 0.02773 | 0.01798 | -0.00290 | 0.00072 | -0.00050 | No |
| 5 | O1 | 0.03417 | 0.02408 | 0.05675 | 0.02174 | -0.00311 | -0.00926 | -0.00199 | No |
| 6 | O2 | 0.04066 | 0.05117 | 0.02891 | 0.04249 | 0.00104 | -0.01337 | -0.01991 | No |
| 7 | O3 | 0.03973 | 0.04715 | 0.04604 | 0.02569 | -0.01451 | -0.01234 | 0.00996 | No |
| 8 | O4 | 0.04480 | 0.04916 | 0.06639 | 0.01877 | -0.00415 | 0.00309 | 0.00199 | No |
| 9 | O5 | 0.03570 | 0.02709 | 0.03748 | 0.04249 | -0.00518 | -0.01337 | 0.00100 | No |
| 10 | O6 | 0.03165 | 0.02909 | 0.04604 | 0.01976 | -0.00311 | -0.00720 | 0.00100 | No |
| 11 | O7 | 0.04124 | 0.03712 | 0.05889 | 0.02767 | -0.00207 | -0.01440 | 0.00100 | No |
| 12 | O8 | 0.04897 | 0.04615 | 0.07923 | 0.02174 | 0.01036 | -0.00103 | -0.00797 | No |
| 13 | O9 | 0.03247 | 0.04515 | 0.03641 | 0.01581 | -0.00622 | 0.00411 | 0.00100 | No |
| 14 | O10 | 0.03740 | 0.03612 | 0.03641 | 0.03952 | -0.00207 | 0.01234 | 0.00498 | No |
| 15 | O11 | 0.03742 | 0.03210 | 0.05354 | 0.02668 | -0.00726 | 0.01131 | -0.00199 | No |
| 16 | O12 | 0.03896 | 0.03812 | 0.05889 | 0.01976 | 0.00829 | -0.00103 | 0.00299 | No |
| 17 | O13 | 0.03696 | 0.03210 | 0.04925 | 0.02964 | 0.00726 | -0.01337 | -0.00498 | No |
| 18 | O14 | 0.04519 | 0.04414 | 0.06853 | 0.02273 | 0.00104 | 0.00720 | 0.00498 | No |
| 19 | O15 | 0.04449 | 0.05417 | 0.03641 | 0.04249 | -0.00415 | -0.01543 | 0.01294 | No |
| 20 | O16 | 0.03597 | 0.02307 | 0.06210 | 0.02273 | 0.00726 | -0.01234 | 0.00000 | No |
| 21 | O17 | 0.05850 | 0.02809 | 0.10493 | 0.04249 | -0.00104 | -0.00103 | -0.00100 | No |
| 22 | O18 | 0.04371 | 0.02207 | 0.05568 | 0.05336 | 0.00000 | -0.02160 | 0.00000 | No |
| 23 | O19 | 0.05845 | 0.03010 | 0.10172 | 0.04348 | 0.00000 | -0.01131 | 0.00100 | No |
| 24 | O20 | 0.03786 | 0.03712 | 0.04176 | 0.03458 | -0.00415 | -0.01029 | 0.00299 | No |
| 25 | O21 | 0.07306 | 0.02608 | 0.14562 | 0.04743 | -0.00311 | 0.01954 | 0.00100 | No |
| 26 | O22 | 0.04388 | 0.03110 | 0.04818 | 0.05237 | -0.00311 | 0.02366 | -0.00100 | No |
| 27 | O23 | 0.06193 | 0.03110 | 0.11243 | 0.04249 | -0.00311 | 0.02057 | -0.00797 | No |
| 28 | O24 | 0.04272 | 0.03110 | 0.05461 | 0.04249 | -0.00829 | 0.01749 | -0.00199 | No |
| 29 | N1 | 0.03137 | 0.03913 | 0.03426 | 0.02075 | -0.00311 | 0.00309 | -0.00100 | No |
| 30 | N2 | 0.04791 | 0.01906 | 0.07067 | 0.05435 | 0.00415 | -0.02057 | -0.01195 | No |
| 31 | H2 | 0.05600 | 0.05600 | 0.05600 | 0.05600 | 0.00000 | 0.00000 | 0.00000 | No |
| 32 | N3 | 0.02998 | 0.02408 | 0.03212 | 0.03360 | 0.00000 | -0.00103 | 0.00398 | No |
| 33 | N4 | 0.02965 | 0.01605 | 0.03641 | 0.03656 | 0.00207 | 0.00103 | -0.00299 | No |
| 34 | N5 | 0.03322 | 0.03511 | 0.04176 | 0.02273 | -0.00104 | 0.00309 | 0.00199 | No |
| 35 | N6 | 0.03582 | 0.02508 | 0.05461 | 0.02767 | 0.00104 | -0.00617 | 0.00299 | No |
| 36 | H6 | 0.04200 | 0.04200 | 0.04200 | 0.04200 | 0.00000 | 0.00000 | 0.00000 | No |
| 37 | N7 | 0.03178 | 0.02909 | 0.03748 | 0.02866 | 0.00207 | -0.00206 | 0.00299 | No |
| 38 | H7 | 0.03700 | 0.03700 | 0.03700 | 0.03700 | 0.00000 | 0.00000 | 0.00000 | No |
| 39 | N8 | 0.01630 | 0.01630 | 0.01630 | 0.01630 | 0.00000 | 0.00000 | 0.00000 | No |
| 40 | N9 | 0.03137 | 0.02408 | 0.05140 | 0.01877 | 0.00000 | -0.00103 | -0.00498 | No |
| 41 | N10 | 0.05155 | 0.02709 | 0.08994 | 0.03755 | -0.00207 | -0.01646 | 0.00199 | No |
| 42 | H10 | 0.06000 | 0.06000 | 0.06000 | 0.06000 | 0.00000 | 0.00000 | 0.00000 | No |
| 43 | N11 | 0.03354 | 0.03210 | 0.04176 | 0.02668 | -0.00104 | 0.00309 | 0.00199 | No |
| 44 | H11 | 0.04000 | 0.04000 | 0.04000 | 0.04000 | 0.00000 | 0.00000 | 0.00000 | No |
| 45 | N12 | 0.03471 | 0.03612 | 0.03319 | 0.03458 | 0.00000 | -0.00514 | 0.00697 | No |
| 46 | N13 | 0.02084 | 0.01204 | 0.02677 | 0.02371 | 0.00000 | 0.01131 | 0.00000 | No |
| 47 | N14 | 0.05744 | 0.02006 | 0.11671 | 0.03557 | -0.00726 | 0.02777 | -0.00100 | No |
| 48 | H14 | 0.06600 | 0.06600 | 0.06600 | 0.06600 | 0.00000 | 0.00000 | 0.00000 | No |
| 49 | N15 | 0.03748 | 0.01304 | 0.04711 | 0.05237 | 0.00000 | 0.00823 | -0.00299 | No |
| 50 | H15 | 0.04400 | 0.04400 | 0.04400 | 0.04400 | 0.00000 | 0.00000 | 0.00000 | No |
| 51 | N16 | 0.02789 | 0.02809 | 0.02784 | 0.02767 | 0.00000 | -0.00411 | 0.00199 | No |
| 52 | C1 | 0.02394 | 0.02307 | 0.03105 | 0.01779 | -0.00104 | -0.00411 | -0.00299 | No |
| 53 | C2 | 0.03578 | 0.03812 | 0.03641 | 0.03261 | -0.00104 | -0.00617 | 0.00597 | No |
| 54 | H2A | 0.04200 | 0.04200 | 0.04200 | 0.04200 | 0.00000 | 0.00000 | 0.00000 | No |
| 55 | C3 | 0.03448 | 0.04013 | 0.03855 | 0.02470 | -0.00933 | -0.00103 | 0.00199 | No |
| 56 | H3 | 0.04000 | 0.04000 | 0.04000 | 0.04000 | 0.00000 | 0.00000 | 0.00000 | No |
| 57 | C4 | 0.02875 | 0.02408 | 0.03748 | 0.02470 | -0.00415 | 0.00103 | -0.00100 | No |
| 58 | C5 | 0.03130 | 0.03511 | 0.03212 | 0.02668 | -0.00415 | 0.00617 | -0.00100 | No |
| 59 | H5 | 0.03700 | 0.03700 | 0.03700 | 0.03700 | 0.00000 | 0.00000 | 0.00000 | No |
| 60 | C6 | 0.03149 | 0.03712 | 0.02677 | 0.03063 | -0.00726 | 0.00206 | -0.00199 | No |
| 61 | H6A | 0.03700 | 0.03700 | 0.03700 | 0.03700 | 0.00000 | 0.00000 | 0.00000 | No |
| 62 | C7 | 0.02985 | 0.02408 | 0.04390 | 0.02174 | 0.00622 | -0.01029 | -0.00597 | No |
| 63 | C8 | 0.03355 | 0.02207 | 0.05782 | 0.02075 | -0.00311 | -0.00206 | 0.00000 | No |
| 64 | C9 | 0.02741 | 0.01906 | 0.03748 | 0.02569 | 0.00622 | -0.00206 | -0.00000 | No |
| 65 | C10 | 0.03224 | 0.03511 | 0.02891 | 0.03261 | -0.00207 | 0.00000 | 0.00299 | No |
| 66 | H10A | 0.03800 | 0.03800 | 0.03800 | 0.03800 | 0.00000 | 0.00000 | 0.00000 | No |
| 67 | C11 | 0.03622 | 0.04615 | 0.04176 | 0.02075 | 0.00104 | 0.00206 | 0.00000 | No |
| 68 | H11A | 0.04200 | 0.04200 | 0.04200 | 0.04200 | 0.00000 | 0.00000 | 0.00000 | No |
| 69 | C12 | 0.02769 | 0.02809 | 0.03426 | 0.02075 | 0.00518 | -0.00206 | -0.00199 | No |
| 70 | C13 | 0.02725 | 0.03511 | 0.02891 | 0.01779 | 0.00104 | -0.00411 | -0.00199 | No |
| 71 | H13 | 0.03200 | 0.03200 | 0.03200 | 0.03200 | 0.00000 | 0.00000 | 0.00000 | No |
| 72 | C14 | 0.02722 | 0.03210 | 0.02891 | 0.02075 | -0.00518 | 0.00103 | -0.00398 | No |
| 73 | H14A | 0.03200 | 0.03200 | 0.03200 | 0.03200 | 0.00000 | 0.00000 | 0.00000 | No |
| 74 | C15 | 0.02978 | 0.01706 | 0.04069 | 0.03162 | 0.00622 | -0.00514 | -0.00100 | No |
| 75 | C16 | 0.03439 | 0.01605 | 0.06639 | 0.02075 | 0.00311 | -0.01337 | -0.00100 | No |
| 76 | C17 | 0.02535 | 0.02408 | 0.03426 | 0.01779 | 0.00415 | -0.00103 | -0.00299 | No |
| 77 | C18 | 0.03169 | 0.03110 | 0.03426 | 0.02964 | 0.00104 | 0.00309 | 0.00199 | No |
| 78 | H18 | 0.03700 | 0.03700 | 0.03700 | 0.03700 | 0.00000 | 0.00000 | 0.00000 | No |
| 79 | C19 | 0.03305 | 0.03612 | 0.03533 | 0.02767 | 0.00207 | -0.00103 | 0.00100 | No |
| 80 | H19 | 0.03900 | 0.03900 | 0.03900 | 0.03900 | 0.00000 | 0.00000 | 0.00000 | No |
| 81 | C20 | 0.02838 | 0.03110 | 0.03319 | 0.02075 | -0.00104 | 0.00720 | 0.00299 | No |
| 82 | C21 | 0.02997 | 0.04113 | 0.03212 | 0.01680 | -0.00933 | 0.00617 | -0.00498 | No |
| 83 | H21 | 0.03500 | 0.03500 | 0.03500 | 0.03500 | 0.00000 | 0.00000 | 0.00000 | No |
| 84 | C22 | 0.03063 | 0.04615 | 0.03105 | 0.01482 | -0.00207 | -0.00411 | -0.00498 | No |
| 85 | H22 | 0.03600 | 0.03600 | 0.03600 | 0.03600 | 0.00000 | 0.00000 | 0.00000 | No |
| 86 | C23 | 0.02970 | 0.02508 | 0.03426 | 0.02964 | 0.00311 | 0.01029 | 0.00299 | No |
| 87 | C24 | 0.02947 | 0.01906 | 0.05354 | 0.01581 | 0.00000 | 0.01131 | -0.00000 | No |
| 88 | C25 | 0.02804 | 0.02107 | 0.03641 | 0.02668 | 0.00207 | 0.00309 | -0.00100 | No |
| 89 | C26 | 0.03324 | 0.04013 | 0.02891 | 0.03063 | 0.00207 | -0.01029 | 0.00100 | No |
| 90 | H26 | 0.03900 | 0.03900 | 0.03900 | 0.03900 | 0.00000 | 0.00000 | 0.00000 | No |
| 91 | C27 | 0.03162 | 0.04314 | 0.03105 | 0.02075 | 0.00000 | 0.00206 | -0.00299 | No |
| 92 | H27 | 0.03700 | 0.03700 | 0.03700 | 0.03700 | 0.00000 | 0.00000 | 0.00000 | No |
| 93 | C28 | 0.02683 | 0.02909 | 0.03855 | 0.01285 | 0.00415 | -0.01029 | 0.00000 | No |
| 94 | C29 | 0.03349 | 0.03010 | 0.02784 | 0.04249 | 0.00415 | 0.00206 | 0.00100 | No |
| 95 | H29 | 0.04000 | 0.04000 | 0.04000 | 0.04000 | 0.00000 | 0.00000 | 0.00000 | No |
| 96 | C30 | 0.03008 | 0.03311 | 0.03533 | 0.02174 | 0.00207 | -0.00103 | 0.00199 | No |
| 97 | H30 | 0.03500 | 0.03500 | 0.03500 | 0.03500 | 0.00000 | 0.00000 | 0.00000 | No |
| 98 | C31 | 0.03157 | 0.01405 | 0.04604 | 0.03458 | 0.00104 | -0.01131 | 0.00100 | No |
| 99 | C32 | 0.02962 | 0.02408 | 0.04390 | 0.02075 | 0.00000 | -0.01543 | 0.00398 | No |
| 100 | C33 | 0.03496 | 0.02608 | 0.04925 | 0.02964 | 0.00311 | -0.00926 | -0.00398 | No |
| 101 | H33 | 0.04100 | 0.04100 | 0.04100 | 0.04100 | 0.00000 | 0.00000 | 0.00000 | No |
| 102 | C34 | 0.03914 | 0.03511 | 0.05461 | 0.02767 | 0.00104 | 0.00103 | 0.00100 | No |
| 103 | H34 | 0.04500 | 0.04500 | 0.04500 | 0.04500 | 0.00000 | 0.00000 | 0.00000 | No |
| 104 | C35 | 0.03733 | 0.02307 | 0.04818 | 0.04051 | -0.00726 | -0.01029 | 0.00697 | No |
| 105 | C36 | 0.05140 | 0.03411 | 0.08352 | 0.03656 | -0.01347 | -0.01440 | -0.00000 | No |
| 106 | H36 | 0.06000 | 0.06000 | 0.06000 | 0.06000 | 0.00000 | 0.00000 | 0.00000 | No |
| 107 | C37 | 0.04697 | 0.02207 | 0.07923 | 0.03952 | 0.00000 | -0.00514 | 0.00199 | No |
| 108 | H37 | 0.05400 | 0.05400 | 0.05400 | 0.05400 | 0.00000 | 0.00000 | 0.00000 | No |
| 109 | C38 | 0.03516 | 0.02107 | 0.04283 | 0.04150 | -0.00311 | -0.00309 | 0.00199 | No |
| 110 | C39 | 0.04042 | 0.02608 | 0.03855 | 0.05632 | 0.00311 | -0.01234 | 0.00996 | No |
| 111 | C40 | 0.04608 | 0.02207 | 0.06639 | 0.04941 | 0.00000 | 0.00309 | 0.01195 | No |
| 112 | H40 | 0.05400 | 0.05400 | 0.05400 | 0.05400 | 0.00000 | 0.00000 | 0.00000 | No |
| 113 | C41 | 0.04811 | 0.03411 | 0.06960 | 0.04051 | -0.00311 | -0.00309 | 0.00299 | No |
| 114 | H41 | 0.05600 | 0.05600 | 0.05600 | 0.05600 | 0.00000 | 0.00000 | 0.00000 | No |
| 115 | C42 | 0.03713 | 0.02909 | 0.02463 | 0.05731 | 0.00415 | 0.00720 | 0.01095 | No |
| 116 | C43 | 0.04316 | 0.02809 | 0.05782 | 0.04348 | 0.00311 | -0.01029 | 0.00299 | No |
| 117 | H43 | 0.05000 | 0.05000 | 0.05000 | 0.05000 | 0.00000 | 0.00000 | 0.00000 | No |
| 118 | C44 | 0.04309 | 0.02408 | 0.05889 | 0.04644 | 0.00000 | -0.01029 | -0.00498 | No |
| 119 | H44 | 0.05000 | 0.05000 | 0.05000 | 0.05000 | 0.00000 | 0.00000 | 0.00000 | No |
| 120 | C45 | 0.03297 | 0.03110 | 0.02249 | 0.04545 | -0.00518 | -0.00103 | -0.00498 | No |
| 121 | C46 | 0.03555 | 0.03210 | 0.04497 | 0.02964 | -0.00207 | -0.00720 | -0.00299 | No |
| 122 | H46 | 0.04200 | 0.04200 | 0.04200 | 0.04200 | 0.00000 | 0.00000 | 0.00000 | No |
| 123 | C47 | 0.03482 | 0.02809 | 0.03962 | 0.03656 | -0.00104 | -0.00823 | 0.00597 | No |
| 124 | H47 | 0.04000 | 0.04000 | 0.04000 | 0.04000 | 0.00000 | 0.00000 | 0.00000 | No |
| 125 | C48 | 0.02682 | 0.02909 | 0.02570 | 0.02569 | 0.00311 | 0.00514 | -0.00100 | No |
| 126 | C49 | 0.03821 | 0.02608 | 0.05996 | 0.02866 | -0.00311 | -0.00720 | -0.00299 | No |
| 127 | H49 | 0.04400 | 0.04400 | 0.04400 | 0.04400 | 0.00000 | 0.00000 | 0.00000 | No |
| 128 | C50 | 0.03887 | 0.03010 | 0.05889 | 0.02767 | -0.00207 | -0.01131 | -0.00199 | No |
| 129 | H50 | 0.04500 | 0.04500 | 0.04500 | 0.04500 | 0.00000 | 0.00000 | 0.00000 | No |
| 130 | C51 | 0.04662 | 0.03511 | 0.07709 | 0.02767 | -0.00622 | -0.01749 | -0.00100 | No |
| 131 | H51 | 0.05400 | 0.05400 | 0.05400 | 0.05400 | 0.00000 | 0.00000 | 0.00000 | No |
| 132 | C52 | 0.04962 | 0.02909 | 0.09101 | 0.02866 | -0.00622 | -0.02571 | 0.00299 | No |
| 133 | H52 | 0.05700 | 0.05700 | 0.05700 | 0.05700 | 0.00000 | 0.00000 | 0.00000 | No |
| 134 | C53 | 0.03549 | 0.02207 | 0.04283 | 0.04150 | 0.00104 | -0.00720 | 0.00199 | No |
| 135 | C54 | 0.04628 | 0.02608 | 0.08031 | 0.03261 | 0.00000 | -0.01131 | -0.00597 | No |
| 136 | H54 | 0.05400 | 0.05400 | 0.05400 | 0.05400 | 0.00000 | 0.00000 | 0.00000 | No |
| 137 | C55 | 0.04602 | 0.02207 | 0.08245 | 0.03360 | -0.00104 | -0.01646 | -0.00199 | No |
| 138 | H55 | 0.05300 | 0.05300 | 0.05300 | 0.05300 | 0.00000 | 0.00000 | 0.00000 | No |
| 139 | C56 | 0.03934 | 0.02608 | 0.05140 | 0.04051 | 0.00518 | 0.00411 | -0.00000 | No |
| 140 | C57 | 0.03498 | 0.02508 | 0.03533 | 0.04447 | 0.00207 | -0.00103 | 0.00100 | No |
| 141 | C58 | 0.04043 | 0.03411 | 0.05032 | 0.03656 | 0.00104 | -0.00309 | 0.00896 | No |
| 142 | H58 | 0.04700 | 0.04700 | 0.04700 | 0.04700 | 0.00000 | 0.00000 | 0.00000 | No |
| 143 | C59 | 0.03648 | 0.02608 | 0.04390 | 0.03952 | -0.00311 | -0.00103 | -0.00299 | No |
| 144 | H59 | 0.04300 | 0.04300 | 0.04300 | 0.04300 | 0.00000 | 0.00000 | 0.00000 | No |
| 145 | C60 | 0.02683 | 0.02408 | 0.02463 | 0.03162 | -0.00104 | 0.00514 | 0.00498 | No |
| 146 | C61 | 0.04516 | 0.02508 | 0.07281 | 0.03755 | -0.00518 | -0.00617 | 0.00100 | No |
| 147 | H61 | 0.05200 | 0.05200 | 0.05200 | 0.05200 | 0.00000 | 0.00000 | 0.00000 | No |
| 148 | C62 | 0.05007 | 0.03210 | 0.07067 | 0.04743 | -0.00207 | 0.00411 | 0.00000 | No |
| 149 | H62 | 0.05900 | 0.05900 | 0.05900 | 0.05900 | 0.00000 | 0.00000 | 0.00000 | No |
| 150 | C63 | 0.03155 | 0.02809 | 0.02998 | 0.03656 | 0.00104 | -0.00514 | 0.00000 | No |
| 151 | C64 | 0.03095 | 0.03210 | 0.04711 | 0.01383 | 0.00518 | -0.00926 | -0.00697 | No |
| 152 | H64 | 0.03600 | 0.03600 | 0.03600 | 0.03600 | 0.00000 | 0.00000 | 0.00000 | No |
| 153 | C65 | 0.03159 | 0.02307 | 0.03533 | 0.03656 | 0.00207 | -0.00411 | -0.00697 | No |
| 154 | H65 | 0.03800 | 0.03800 | 0.03800 | 0.03800 | 0.00000 | 0.00000 | 0.00000 | No |
| 155 | C66 | 0.02924 | 0.01906 | 0.03212 | 0.03656 | 0.00000 | 0.01131 | -0.00100 | No |
| 156 | C67 | 0.03512 | 0.03110 | 0.04069 | 0.03360 | 0.00518 | -0.00926 | -0.00100 | No |
| 157 | H67 | 0.04100 | 0.04100 | 0.04100 | 0.04100 | 0.00000 | 0.00000 | 0.00000 | No |
| 158 | C68 | 0.03006 | 0.02909 | 0.03855 | 0.02273 | 0.00104 | 0.00206 | -0.00697 | No |
| 159 | H68 | 0.03500 | 0.03500 | 0.03500 | 0.03500 | 0.00000 | 0.00000 | 0.00000 | No |
| 160 | C69 | 0.04300 | 0.04300 | 0.04300 | 0.04300 | 0.00000 | 0.00000 | 0.00000 | No |
| 161 | H69 | 0.05200 | 0.05200 | 0.05200 | 0.05200 | 0.00000 | 0.00000 | 0.00000 | No |
| 162 | C70 | 0.04800 | 0.04800 | 0.04800 | 0.04800 | 0.00000 | 0.00000 | 0.00000 | No |
| 163 | H70 | 0.05800 | 0.05800 | 0.05800 | 0.05800 | 0.00000 | 0.00000 | 0.00000 | No |
| 164 | C69' | 0.01900 | 0.01900 | 0.01900 | 0.01900 | 0.00000 | 0.00000 | 0.00000 | No |
| 165 | H69' | 0.02300 | 0.02300 | 0.02300 | 0.02300 | 0.00000 | 0.00000 | 0.00000 | No |
| 166 | C70' | 0.02600 | 0.02600 | 0.02600 | 0.02600 | 0.00000 | 0.00000 | 0.00000 | No |
| 167 | H70' | 0.03200 | 0.03200 | 0.03200 | 0.03200 | 0.00000 | 0.00000 | 0.00000 | No |
| 168 | C71 | 0.04776 | 0.01405 | 0.09101 | 0.03854 | 0.00311 | -0.00206 | -0.01095 | No |
| 169 | C72 | 0.04500 | 0.04500 | 0.04500 | 0.04500 | 0.00000 | 0.00000 | 0.00000 | No |
| 170 | H72 | 0.05400 | 0.05400 | 0.05400 | 0.05400 | 0.00000 | 0.00000 | 0.00000 | No |
| 171 | C73 | 0.04000 | 0.04000 | 0.04000 | 0.04000 | 0.00000 | 0.00000 | 0.00000 | No |
| 172 | H73 | 0.04800 | 0.04800 | 0.04800 | 0.04800 | 0.00000 | 0.00000 | 0.00000 | No |
| 173 | C72' | 0.04100 | 0.04100 | 0.04100 | 0.04100 | 0.00000 | 0.00000 | 0.00000 | No |
| 174 | H72' | 0.05000 | 0.05000 | 0.05000 | 0.05000 | 0.00000 | 0.00000 | 0.00000 | No |
| 175 | C73' | 0.03100 | 0.03100 | 0.03100 | 0.03100 | 0.00000 | 0.00000 | 0.00000 | No |
| 176 | H73' | 0.03700 | 0.03700 | 0.03700 | 0.03700 | 0.00000 | 0.00000 | 0.00000 | No |
| 177 | C74 | 0.04534 | 0.03110 | 0.06746 | 0.03755 | 0.00104 | -0.00206 | -0.00299 | No |
| 178 | C75 | 0.04745 | 0.02508 | 0.07709 | 0.04051 | 0.00104 | -0.01543 | -0.01195 | No |
| 179 | C76 | 0.03800 | 0.03800 | 0.03800 | 0.03800 | 0.00000 | 0.00000 | 0.00000 | No |
| 180 | H76 | 0.04600 | 0.04600 | 0.04600 | 0.04600 | 0.00000 | 0.00000 | 0.00000 | No |
| 181 | C77 | 0.03600 | 0.03600 | 0.03600 | 0.03600 | 0.00000 | 0.00000 | 0.00000 | No |
| 182 | H77 | 0.04400 | 0.04400 | 0.04400 | 0.04400 | 0.00000 | 0.00000 | 0.00000 | No |
| 183 | C76' | 0.04600 | 0.04600 | 0.04600 | 0.04600 | 0.00000 | 0.00000 | 0.00000 | No |
| 184 | H76' | 0.05500 | 0.05500 | 0.05500 | 0.05500 | 0.00000 | 0.00000 | 0.00000 | No |
| 185 | C77' | 0.04400 | 0.04400 | 0.04400 | 0.04400 | 0.00000 | 0.00000 | 0.00000 | No |
| 186 | H77' | 0.05300 | 0.05300 | 0.05300 | 0.05300 | 0.00000 | 0.00000 | 0.00000 | No |
| 187 | C78 | 0.03854 | 0.03010 | 0.03105 | 0.05435 | -0.00415 | -0.01440 | 0.00398 | No |
| 188 | C79 | 0.03500 | 0.03500 | 0.03500 | 0.03500 | 0.00000 | 0.00000 | 0.00000 | No |
| 189 | H79 | 0.04200 | 0.04200 | 0.04200 | 0.04200 | 0.00000 | 0.00000 | 0.00000 | No |
| 190 | C80 | 0.03800 | 0.03800 | 0.03800 | 0.03800 | 0.00000 | 0.00000 | 0.00000 | No |
| 191 | H80 | 0.04500 | 0.04500 | 0.04500 | 0.04500 | 0.00000 | 0.00000 | 0.00000 | No |
| 192 | C79' | 0.03800 | 0.03800 | 0.03800 | 0.03800 | 0.00000 | 0.00000 | 0.00000 | No |
| 193 | H79' | 0.04500 | 0.04500 | 0.04500 | 0.04500 | 0.00000 | 0.00000 | 0.00000 | No |
| 194 | C80' | 0.03800 | 0.03800 | 0.03800 | 0.03800 | 0.00000 | 0.00000 | 0.00000 | No |
| 195 | H80' | 0.04500 | 0.04500 | 0.04500 | 0.04500 | 0.00000 | 0.00000 | 0.00000 | No |
| 196 | C81 | 0.03243 | 0.03511 | 0.02356 | 0.03854 | 0.00000 | 0.00514 | 0.00199 | No |
| 197 | C82 | 0.03048 | 0.02608 | 0.03748 | 0.02767 | 0.00518 | -0.00103 | 0.00697 | No |
| 198 | H82 | 0.03600 | 0.03600 | 0.03600 | 0.03600 | 0.00000 | 0.00000 | 0.00000 | No |
| 199 | C83 | 0.03206 | 0.03712 | 0.03748 | 0.02174 | 0.00518 | 0.00411 | -0.00597 | No |
| 200 | H83 | 0.03800 | 0.03800 | 0.03800 | 0.03800 | 0.00000 | 0.00000 | 0.00000 | No |
| 201 | C84 | 0.02921 | 0.04214 | 0.02463 | 0.02075 | 0.00104 | -0.00720 | 0.00299 | No |
| 202 | C85 | 0.03430 | 0.03612 | 0.04283 | 0.02371 | -0.00104 | -0.00103 | 0.00797 | No |
| 203 | H85 | 0.04000 | 0.04000 | 0.04000 | 0.04000 | 0.00000 | 0.00000 | 0.00000 | No |
| 204 | C86 | 0.03256 | 0.03411 | 0.04390 | 0.01976 | -0.00104 | 0.00206 | -0.00398 | No |
| 205 | H86 | 0.03800 | 0.03800 | 0.03800 | 0.03800 | 0.00000 | 0.00000 | 0.00000 | No |
| 206 | C87 | 0.03523 | 0.03010 | 0.04390 | 0.03162 | 0.00311 | 0.00514 | 0.00199 | No |
| 207 | H87 | 0.04100 | 0.04100 | 0.04100 | 0.04100 | 0.00000 | 0.00000 | 0.00000 | No |
| 208 | C88 | 0.03935 | 0.02408 | 0.05461 | 0.03952 | 0.00311 | 0.00309 | -0.00597 | No |
| 209 | H88 | 0.04600 | 0.04600 | 0.04600 | 0.04600 | 0.00000 | 0.00000 | 0.00000 | No |
| 210 | C89 | 0.04771 | 0.01806 | 0.07174 | 0.05336 | 0.00415 | 0.01543 | -0.00100 | No |
| 211 | C90 | 0.03200 | 0.03200 | 0.03200 | 0.03200 | 0.00000 | 0.00000 | 0.00000 | No |
| 212 | C91 | 0.02800 | 0.02800 | 0.02800 | 0.02800 | 0.00000 | 0.00000 | 0.00000 | No |
| 213 | C90' | 0.03500 | 0.03500 | 0.03500 | 0.03500 | 0.00000 | 0.00000 | 0.00000 | No |
| 214 | C91' | 0.03500 | 0.03500 | 0.03500 | 0.03500 | 0.00000 | 0.00000 | 0.00000 | No |
| 215 | C92 | 0.04425 | 0.03110 | 0.06210 | 0.03952 | 0.00000 | 0.00720 | 0.00000 | No |
| 216 | C93 | 0.05138 | 0.01605 | 0.06210 | 0.07608 | -0.00207 | 0.00103 | -0.00398 | No |
| 217 | C94 | 0.04200 | 0.04200 | 0.04200 | 0.04200 | 0.00000 | 0.00000 | 0.00000 | No |
| 218 | H94 | 0.05100 | 0.05100 | 0.05100 | 0.05100 | 0.00000 | 0.00000 | 0.00000 | No |
| 219 | C95 | 0.04500 | 0.04500 | 0.04500 | 0.04500 | 0.00000 | 0.00000 | 0.00000 | No |
| 220 | H95 | 0.05400 | 0.05400 | 0.05400 | 0.05400 | 0.00000 | 0.00000 | 0.00000 | No |
| 221 | C94' | 0.02000 | 0.02000 | 0.02000 | 0.02000 | 0.00000 | 0.00000 | 0.00000 | No |
| 222 | H94' | 0.02300 | 0.02300 | 0.02300 | 0.02300 | 0.00000 | 0.00000 | 0.00000 | No |
| 223 | C95' | 0.01900 | 0.01900 | 0.01900 | 0.01900 | 0.00000 | 0.00000 | 0.00000 | No |
| 224 | H95' | 0.02300 | 0.02300 | 0.02300 | 0.02300 | 0.00000 | 0.00000 | 0.00000 | No |
| 225 | C96 | 0.03429 | 0.02608 | 0.03855 | 0.03854 | -0.00311 | 0.00000 | -0.01095 | No |
| 226 | C97 | 0.03900 | 0.03900 | 0.03900 | 0.03900 | 0.00000 | 0.00000 | 0.00000 | No |
| 227 | H97 | 0.04700 | 0.04700 | 0.04700 | 0.04700 | 0.00000 | 0.00000 | 0.00000 | No |
| 228 | C98 | 0.03300 | 0.03300 | 0.03300 | 0.03300 | 0.00000 | 0.00000 | 0.00000 | No |
| 229 | H98 | 0.04000 | 0.04000 | 0.04000 | 0.04000 | 0.00000 | 0.00000 | 0.00000 | No |
| 230 | C97' | 0.03500 | 0.03500 | 0.03500 | 0.03500 | 0.00000 | 0.00000 | 0.00000 | No |
| 231 | H97' | 0.04200 | 0.04200 | 0.04200 | 0.04200 | 0.00000 | 0.00000 | 0.00000 | No |
| 232 | C98' | 0.03800 | 0.03800 | 0.03800 | 0.03800 | 0.00000 | 0.00000 | 0.00000 | No |
| 233 | H98' | 0.04500 | 0.04500 | 0.04500 | 0.04500 | 0.00000 | 0.00000 | 0.00000 | No |
| 234 | C99 | 0.03436 | 0.02006 | 0.03962 | 0.04348 | 0.00000 | -0.00309 | -0.00299 | No |
| 235 | C100 | 0.03137 | 0.03010 | 0.04818 | 0.01581 | 0.00000 | 0.00514 | 0.00000 | No |
| 236 | H100 | 0.03600 | 0.03600 | 0.03600 | 0.03600 | 0.00000 | 0.00000 | 0.00000 | No |
| 237 | C101 | 0.03529 | 0.03311 | 0.04818 | 0.02470 | 0.00311 | 0.00823 | -0.00498 | No |
| 238 | H101 | 0.04100 | 0.04100 | 0.04100 | 0.04100 | 0.00000 | 0.00000 | 0.00000 | No |
| 239 | C102 | 0.03090 | 0.02909 | 0.02891 | 0.03458 | -0.00415 | -0.00103 | 0.00299 | No |
| 240 | C103 | 0.03027 | 0.02709 | 0.04390 | 0.01976 | -0.00207 | 0.00206 | 0.00199 | No |
| 241 | H103 | 0.03500 | 0.03500 | 0.03500 | 0.03500 | 0.00000 | 0.00000 | 0.00000 | No |
| 242 | C104 | 0.03057 | 0.02207 | 0.04604 | 0.02371 | -0.00311 | 0.00720 | -0.00398 | No |
| 243 | H104 | 0.03600 | 0.03600 | 0.03600 | 0.03600 | 0.00000 | 0.00000 | 0.00000 | No |
| 244 | O1S | 0.06172 | 0.07324 | 0.06532 | 0.04644 | -0.00104 | -0.00514 | 0.00498 | No |
| 245 | N1S | 0.04931 | 0.05016 | 0.06210 | 0.03557 | -0.00726 | 0.00514 | 0.00299 | No |
| 246 | C1S | 0.08106 | 0.05518 | 0.13277 | 0.05533 | -0.00518 | 0.00411 | -0.00398 | No |
| 247 | H1S | 0.09400 | 0.09400 | 0.09400 | 0.09400 | 0.00000 | 0.00000 | 0.00000 | No |
| 248 | C2S | 0.09577 | 0.05819 | 0.16704 | 0.06225 | -0.02695 | 0.04012 | -0.00597 | No |
| 249 | H2S1 | 0.13800 | 0.13800 | 0.13800 | 0.13800 | 0.00000 | 0.00000 | 0.00000 | No |
| 250 | H2S2 | 0.13800 | 0.13800 | 0.13800 | 0.13800 | 0.00000 | 0.00000 | 0.00000 | No |
| 251 | H2S3 | 0.13800 | 0.13800 | 0.13800 | 0.13800 | 0.00000 | 0.00000 | 0.00000 | No |
| 252 | C3S | 0.09372 | 0.06621 | 0.11992 | 0.09486 | 0.00104 | -0.02057 | 0.00398 | No |
| 253 | H3S1 | 0.13700 | 0.13700 | 0.13700 | 0.13700 | 0.00000 | 0.00000 | 0.00000 | No |
| 254 | H3S2 | 0.13700 | 0.13700 | 0.13700 | 0.13700 | 0.00000 | 0.00000 | 0.00000 | No |
| 255 | H3S3 | 0.13700 | 0.13700 | 0.13700 | 0.13700 | 0.00000 | 0.00000 | 0.00000 | No |
| 256 | O2S | 0.06653 | 0.08728 | 0.06424 | 0.04842 | 0.00518 | 0.01440 | -0.01294 | No |
| 257 | N2S | 0.05146 | 0.04113 | 0.07495 | 0.03854 | -0.01451 | -0.00720 | -0.00896 | No |
| 258 | C4S | 0.09397 | 0.07424 | 0.13920 | 0.06818 | -0.00415 | -0.00720 | 0.00896 | No |
| 259 | H4S | 0.10900 | 0.10900 | 0.10900 | 0.10900 | 0.00000 | 0.00000 | 0.00000 | No |
| 260 | C5S | 0.22899 | 0.06220 | 0.42830 | 0.19762 | -0.03420 | 0.23658 | -0.04082 | No |
| 261 | H5S1 | 0.33300 | 0.33300 | 0.33300 | 0.33300 | 0.00000 | 0.00000 | 0.00000 | No |
| 262 | H5S2 | 0.33300 | 0.33300 | 0.33300 | 0.33300 | 0.00000 | 0.00000 | 0.00000 | No |
| 263 | H5S3 | 0.33300 | 0.33300 | 0.33300 | 0.33300 | 0.00000 | 0.00000 | 0.00000 | No |
| 264 | C6S | 0.13792 | 0.05618 | 0.29981 | 0.05731 | -0.04146 | -0.03909 | 0.01493 | No |
| 265 | H6S1 | 0.19800 | 0.19800 | 0.19800 | 0.19800 | 0.00000 | 0.00000 | 0.00000 | No |
| 266 | H6S2 | 0.19800 | 0.19800 | 0.19800 | 0.19800 | 0.00000 | 0.00000 | 0.00000 | No |
| 267 | H6S3 | 0.19800 | 0.19800 | 0.19800 | 0.19800 | 0.00000 | 0.00000 | 0.00000 | No |
| 268 | O3S | 0.12213 | 0.13845 | 0.13277 | 0.09486 | -0.01555 | -0.00103 | 0.00896 | No |
| 269 | N3S | 0.12971 | 0.08929 | 0.08994 | 0.20750 | -0.01347 | -0.01029 | 0.07866 | No |
| 270 | C7S | 0.21484 | 0.25081 | 0.28910 | 0.10869 | 0.16583 | -0.12343 | -0.13939 | No |
| 271 | H7S | 0.25700 | 0.25700 | 0.25700 | 0.25700 | 0.00000 | 0.00000 | 0.00000 | No |
| 272 | C8S | 0.16109 | 0.12039 | 0.21415 | 0.14822 | -0.01036 | -0.07200 | 0.01493 | No |
| 273 | H8S1 | 0.23800 | 0.23800 | 0.23800 | 0.23800 | 0.00000 | 0.00000 | 0.00000 | No |
| 274 | H8S2 | 0.23800 | 0.23800 | 0.23800 | 0.23800 | 0.00000 | 0.00000 | 0.00000 | No |
| 275 | H8S3 | 0.23800 | 0.23800 | 0.23800 | 0.23800 | 0.00000 | 0.00000 | 0.00000 | No |
| 276 | C9S | 0.12263 | 0.10835 | 0.12528 | 0.13537 | -0.01555 | -0.01131 | -0.03883 | No |
| 277 | H9S1 | 0.18200 | 0.18200 | 0.18200 | 0.18200 | 0.00000 | 0.00000 | 0.00000 | No |
| 278 | H9S2 | 0.18200 | 0.18200 | 0.18200 | 0.18200 | 0.00000 | 0.00000 | 0.00000 | No |
| 279 | H9S3 | 0.18200 | 0.18200 | 0.18200 | 0.18200 | 0.00000 | 0.00000 | 0.00000 | No |
| 280 | O4S | 0.15400 | 0.15400 | 0.15400 | 0.15400 | 0.00000 | 0.00000 | 0.00000 | No |
| 281 | N4S | 0.11100 | 0.11100 | 0.11100 | 0.11100 | 0.00000 | 0.00000 | 0.00000 | No |
| 282 | C10S | 0.13200 | 0.13200 | 0.13200 | 0.13200 | 0.00000 | 0.00000 | 0.00000 | No |
| 283 | H10S | 0.15800 | 0.15800 | 0.15800 | 0.15800 | 0.00000 | 0.00000 | 0.00000 | No |
| 284 | C11S | 0.18800 | 0.18800 | 0.18800 | 0.18800 | 0.00000 | 0.00000 | 0.00000 | No |
| 285 | H11B | 0.28100 | 0.28100 | 0.28100 | 0.28100 | 0.00000 | 0.00000 | 0.00000 | No |
| 286 | H11C | 0.28100 | 0.28100 | 0.28100 | 0.28100 | 0.00000 | 0.00000 | 0.00000 | No |
| 287 | H11D | 0.28100 | 0.28100 | 0.28100 | 0.28100 | 0.00000 | 0.00000 | 0.00000 | No |
| 288 | C12S | 0.22300 | 0.22300 | 0.22300 | 0.22300 | 0.00000 | 0.00000 | 0.00000 | No |
| 289 | H12A | 0.33500 | 0.33500 | 0.33500 | 0.33500 | 0.00000 | 0.00000 | 0.00000 | No |
| 290 | H12B | 0.33500 | 0.33500 | 0.33500 | 0.33500 | 0.00000 | 0.00000 | 0.00000 | No |
| 291 | H12C | 0.33500 | 0.33500 | 0.33500 | 0.33500 | 0.00000 | 0.00000 | 0.00000 | No |
| 292 | O5S | 0.15200 | 0.15200 | 0.15200 | 0.15200 | 0.00000 | 0.00000 | 0.00000 | No |
| 293 | N5S | 0.08500 | 0.08500 | 0.08500 | 0.08500 | 0.00000 | 0.00000 | 0.00000 | No |
| 294 | C13S | 0.16900 | 0.16900 | 0.16900 | 0.16900 | 0.00000 | 0.00000 | 0.00000 | No |
| 295 | H13S | 0.20200 | 0.20200 | 0.20200 | 0.20200 | 0.00000 | 0.00000 | 0.00000 | No |
| 296 | C14S | 0.15800 | 0.15800 | 0.15800 | 0.15800 | 0.00000 | 0.00000 | 0.00000 | No |
| 297 | H14B | 0.23800 | 0.23800 | 0.23800 | 0.23800 | 0.00000 | 0.00000 | 0.00000 | No |
| 298 | H14C | 0.23800 | 0.23800 | 0.23800 | 0.23800 | 0.00000 | 0.00000 | 0.00000 | No |
| 299 | H14D | 0.23800 | 0.23800 | 0.23800 | 0.23800 | 0.00000 | 0.00000 | 0.00000 | No |
| 300 | C15S | 0.19200 | 0.19200 | 0.19200 | 0.19200 | 0.00000 | 0.00000 | 0.00000 | No |
| 301 | H15A | 0.28800 | 0.28800 | 0.28800 | 0.28800 | 0.00000 | 0.00000 | 0.00000 | No |
| 302 | H15B | 0.28800 | 0.28800 | 0.28800 | 0.28800 | 0.00000 | 0.00000 | 0.00000 | No |
| 303 | H15C | 0.28800 | 0.28800 | 0.28800 | 0.28800 | 0.00000 | 0.00000 | 0.00000 | No |
| 304 | O6S | 0.11200 | 0.11200 | 0.11200 | 0.11200 | 0.00000 | 0.00000 | 0.00000 | No |
| 305 | O7S | 0.10800 | 0.10800 | 0.10800 | 0.10800 | 0.00000 | 0.00000 | 0.00000 | No |
| 306 | O8S | 0.18800 | 0.18800 | 0.18800 | 0.18800 | 0.00000 | 0.00000 | 0.00000 | No |
| 307 | H | 0.00000 | 0.00000 | 0.00000 | 0.00000 | 0.00000 | 0.00000 | 0.00000 | No |
| 308 | H | 0.00000 | 0.00000 | 0.00000 | 0.00000 | 0.00000 | 0.00000 | 0.00000 | No |
| 309 | H | 0.00000 | 0.00000 | 0.00000 | 0.00000 | 0.00000 | 0.00000 | 0.00000 | No |
| 310 | H | 0.00000 | 0.00000 | 0.00000 | 0.00000 | 0.00000 | 0.00000 | 0.00000 | No |
| 311 | H | 0.00000 | 0.00000 | 0.00000 | 0.00000 | 0.00000 | 0.00000 | 0.00000 | No |

---

### Pattern Parameters

|  |  |  |  |
| --- | --- | --- | --- |
| Profile Function: | Pseudo-Voigt |  |  |

#### FWHM

| Parameter | Value | Refined? |
| --- | --- | --- |
| U | 0.49483 ± *0.36570* | Yes |
| V | 0.04856 ± *0.10243* | Yes |
| W | 0.02584 ± *0.00647* | Yes |

#### Profile

| Parameter | Value | Refined? |
| --- | --- | --- |
| NA | 0.03133 ± *0.07673* | Yes |
| NB | -0.00070 ± *0.00468* | Yes |

#### Line Shift

|  |  |  |  |
| --- | --- | --- | --- |
| Instrument Geometry: | Bragg-Brentano |  |  |

| Parameter | Value | Refined? |
| --- | --- | --- |
| Zero Point | 0.04892 ± *0.00405* | Yes |
| Shift #1 | 0.00000 | No |
| Shift #2 | 0.00000 | No |

---

### Sample Parameters

#### Preferred Orientation

|  |  |  |  |
| --- | --- | --- | --- |
| Function: | March-Dollase |  |  |

| Parameter | Value | Refined? |
| --- | --- | --- |
| a\* | -0.84211 ± *0.02022* | Yes |
| b\* | -0.00012 ± *0.17786* | Yes |
| c\* | -0.53930 ± *0.03157* | Yes |
| R0 | 1.12402 ± *0.01344* | Yes |
