# Supplementary material for: Electro-oxidation sensing of sumatriptan in aqueous solutions and human blood serum by Zn(II)-MOF modified electrochemical delaminated pencil graphite electrode
Source: Sci Rep. 2023 Oct 5;13:16803. doi: 10.1038/s41598-023-44034-5 (PMC10556131; doi:10.1038/s41598-023-44034-5)
Supplement: Supplementary file 2 — Supplementary Information 2. [file 41598_2023_44034_MOESM2_ESM.docx]

**Supplementary information for**

**Electro-oxidation sensing of Sumatriptan in aqueous solutions and human blood serum by Zn(II)-MOF modified electrochemical delaminated pencil graphite electrode**

Lotfali Saghatforoush ^a*^, Tohid Mahmoudi ^a^, Zeynab Khorablou ^b^, Hassan Nasiri ^c^, Akbar Bakhtiari ^a^, [Seyed Ali Akbar](https://www.researchgate.net/profile/Seyed-Ali-Akbar-Sajadi-2) Sajadi ^b^

^a^ Department of Chemistry, Payame Noor University, 19395-4697, Tehran, Iran

^b^ Sharif University of Technology, Sharif Energy, Water and Environment Institute (SEWEI) P.O.Box 11155-8639 Tehran, Iran

^c^ Department of Electrical and Computer Engineering, University of Tabriz, Tabriz, Iran

* Corresponding author:

E-mail address: [saghatforoush@pnu.ac.ir](mailto:saghatforoush@pnu.ac.ir)

[saghatforoush@gmail.com](mailto:saghatforoush@gmail.com)

Tel.: + 98 9141614543

**Material Safety Data Sheet (MSDS).** The properties and possible risks of the materials used in this study are briefly described below.

- **2-amino-1,4-benzene dicarboxylic acid (NH_2_BDC):** Skin corrosion/irritation, serious eye damage/eye irritation
- **ZnCl_2_:** Harmful if swallowed, causes severe skin burns and eye damage, causes serious eye damage, may cause respiratory irritation, very toxic to aquatic life with long lasting effects
- **DMF:** Harmful in contact with skin or if inhaled, causes serious eye irritation, may damage the unborn child, lower and upper explosion limit
- **NaOH:** Causes eye and skin burns, causes digestive and respiratory tract burns.

**
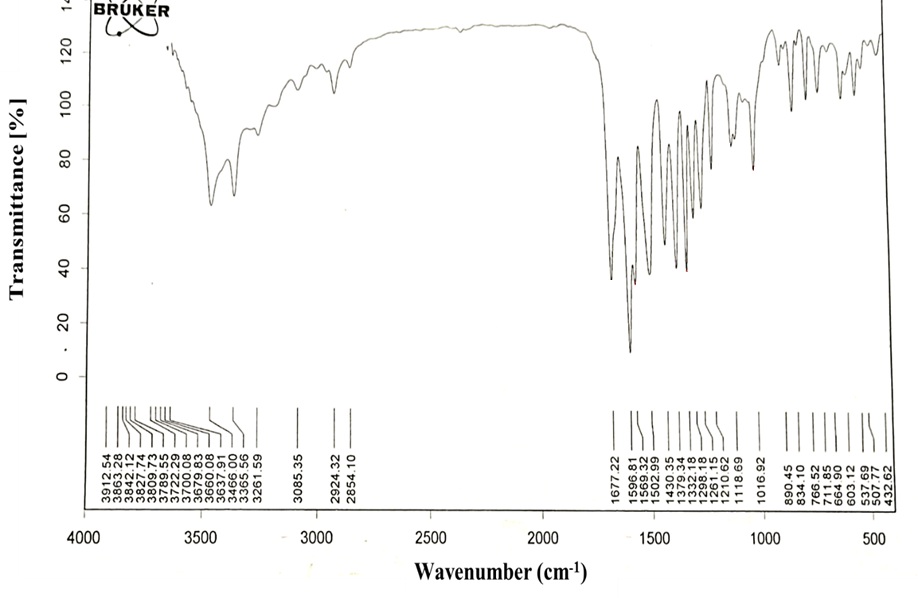
**

**Fig. S1** FTIR spectrum of the synthesized Zn(II)-MOF


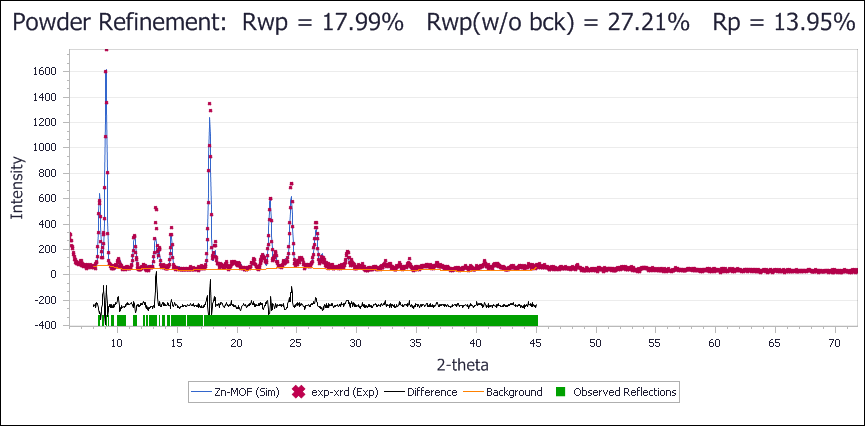


**Fig. S2** The Rietveld plot for the refinement of Zn(II)-MOF. Red crosses: the observed data points; Solid blue line: the calculated pattern; Orange line: the background; Black curve: the difference plot; The row of green tick marks: the observed reflection positions.


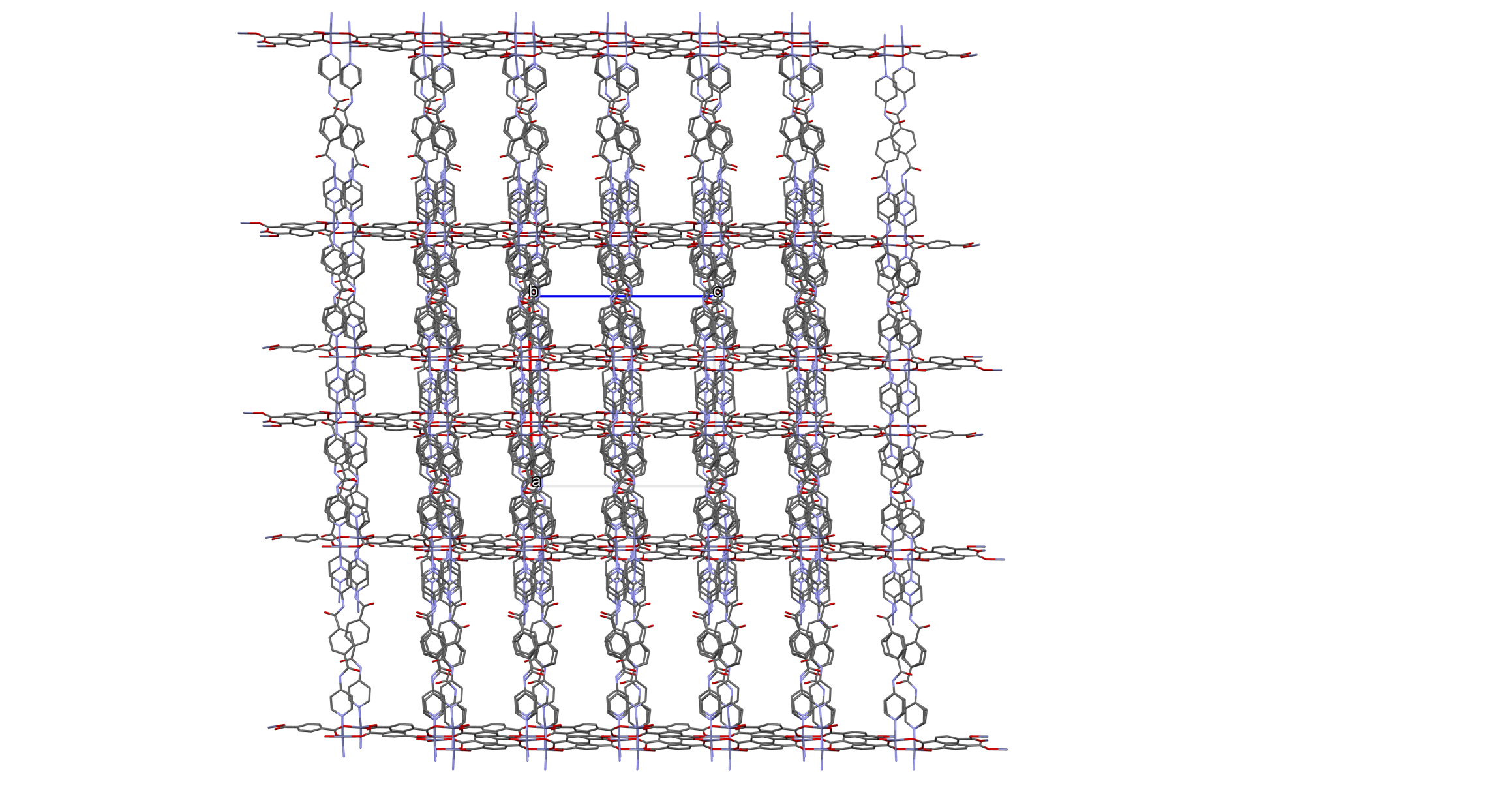


**Fig. S3** The crystal packing of Zn(II)-MOF, viewed along the ***b*** axis. Hydrogen atoms and solvent molecules are not shown for clarity.


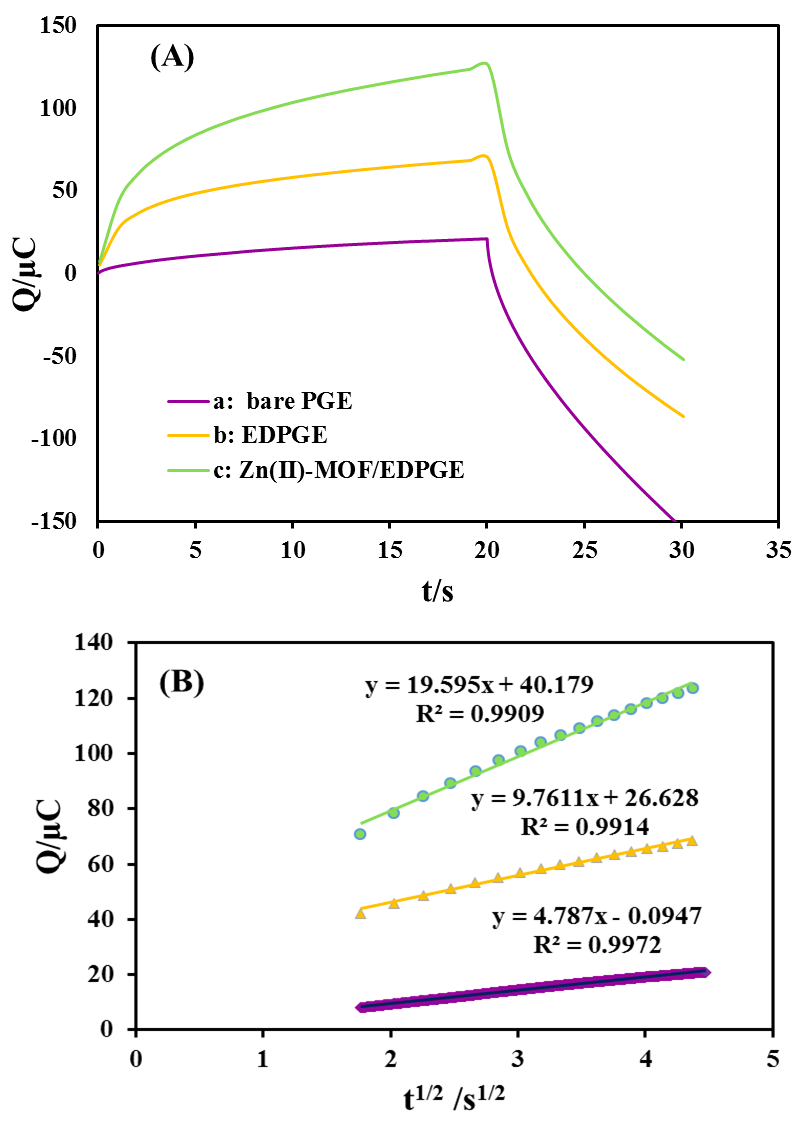


Fig. S4 (A) Q-t and (B) Q-t^1/2^ diagrams for (a) bare PGE (b) EDPGE (c) Zn(II)-MOF/EDPGE


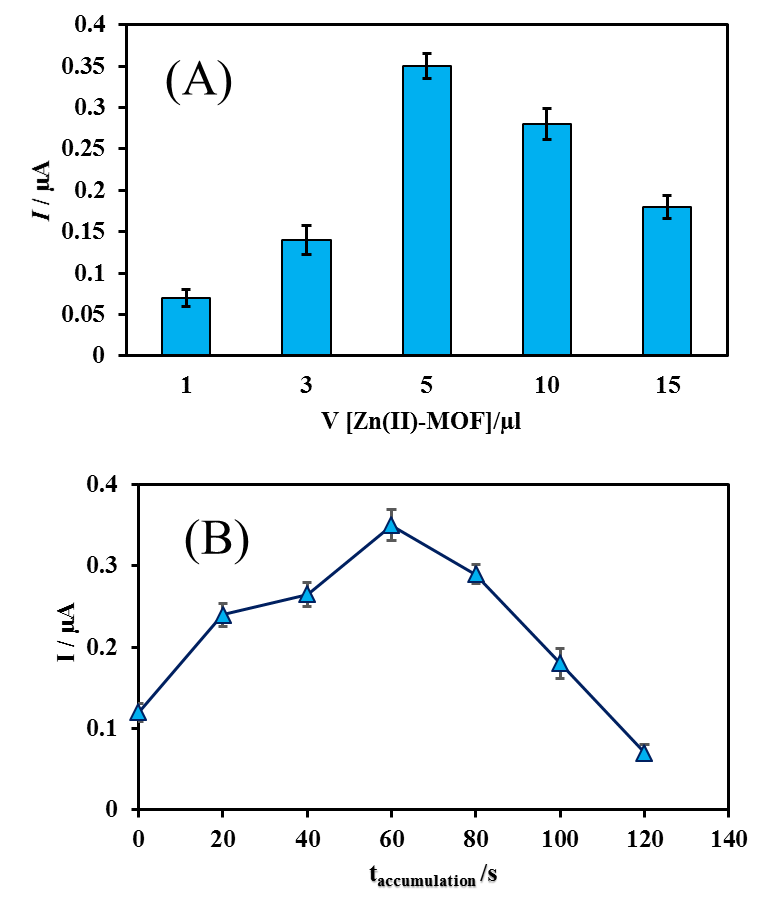


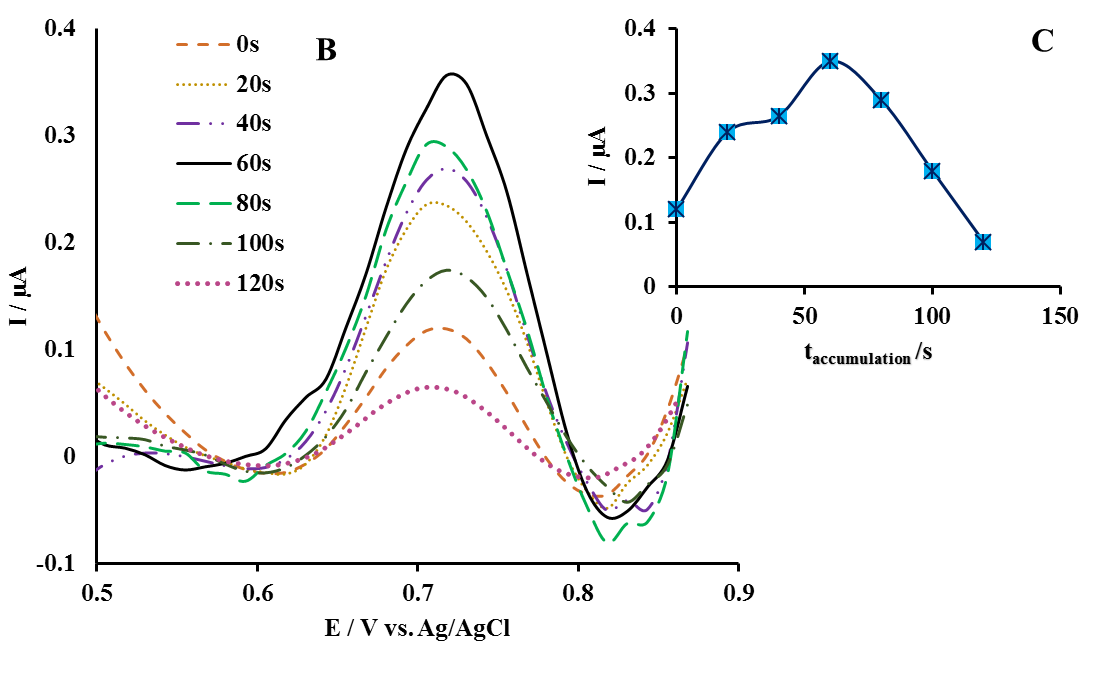


**Fig. S5** **(A)** The anodic peak current changes by the volume of water dispersed Zn(II)-MOF, casted on EDPGE; **(B)** Effect of accumulation time on DPVs measured for 5.82 µM SUM solutions using Zn(II)-MOF/EDPGE; (**C**) Effect of accumulation time on the electro-oxidation current of SUM measured using Zn(II)-MOF/EDPGE. Supporting electrolyte: phosphate buffer, pH = 7.0, scan rate = 20 mV s^-1^.


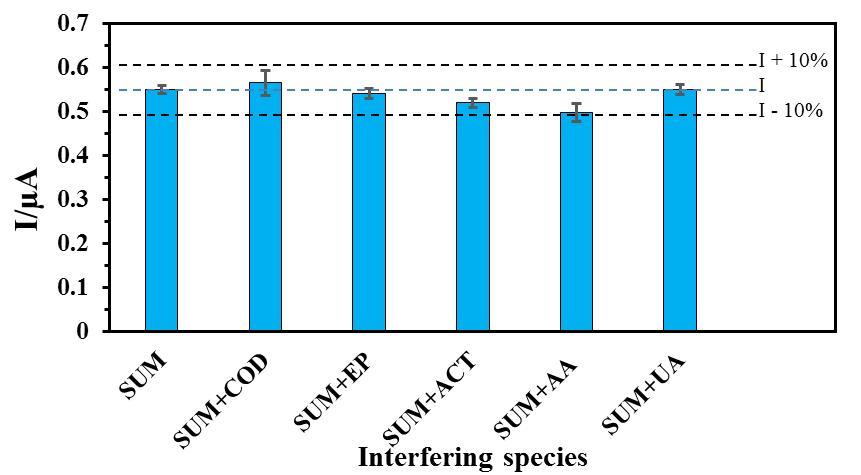


**Fig. S6** The selectivity of Zn(II)-MOF/EDPGE to Sumatriptan (SUM) in the presence of codeine (COD), epinephrine (EP), acetaminophen (ACT), ascorbic acid (AA), and uric acid (UA) (the concentration are 38.45 μM for interfering substances and 7.70 μM of SUM)


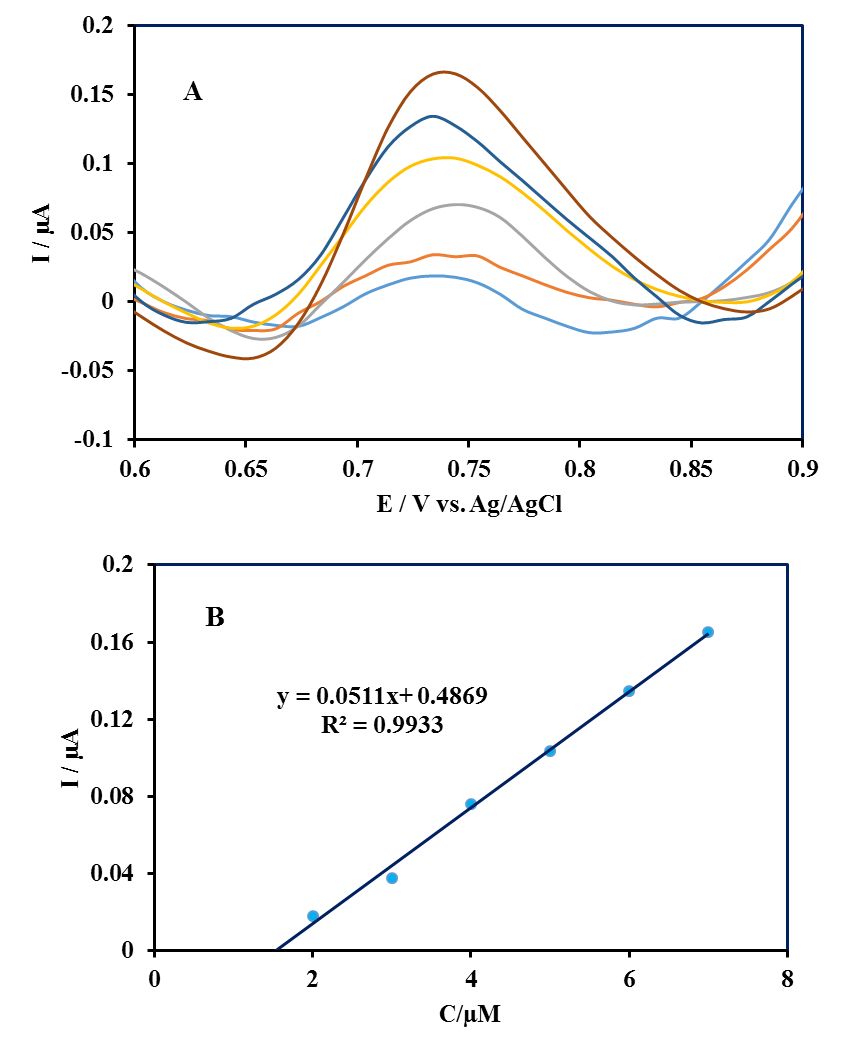


**Fig. S7** The DPVs and corresponding calibration curves for the real samples.
